# CroCoNet: a framework for the quantitative comparison of gene regulatory networks across species

**DOI:** 10.1186/s13059-026-04152-5

**Published:** 2026-07-15

**Authors:** Anita Térmeg, Vladyslav Storozhuk, Zane Kliesmete, Fiona C. Edenhofer, Johanna Geuder, Tamina Dietl, Beate Vieth, Philipp Janssen, Daniel Richter, Boyan Bonev, Ines Hellmann

**Affiliations:** 1 Anthropology and Human Genomics, Ludwig-Maximilians-Universität München, 82152, Planegg, Germany; 2 Research Unit Brain Epigenomics, Helmholtz Center Munich, 81377, Munich, Germany

## Abstract

To understand phenotypic evolution, it is essential to investigate the underlying gene regulatory networks (GRNs). However, most comparative GRN analyzes remain descriptive due to the low signal-to-noise ratio inherent in single-cell transcriptomics data. To address this, we introduce CroCoNet (Cross-species Comparison of Networks), an R-package for quantitative GRN comparison across species. CroCoNet builds comparable network modules centered on putative regulators and compares module topologies within and between species, distinguishing true evolutionary divergence from technical and biological confounders. We demonstrate its utility by comparing early neural differentiation across primates and validating results with a CRISPRi analysis of the diverged *POU5F1* module.

## Background

Linking genotype to phenotype is a central goal in evolutionary biology. Gene regulatory networks (GRNs), which coordinate transcriptional programs across the genome, are key intermediaries in this relationship. Studying GRNs at the level of co-expression modules provides a powerful abstraction: modules often reflect shared regulatory inputs or common functional roles, and can be more conserved across species than individual genes [1, 2]. Module-based comparative analyses of GRNs therefore offer insights into the molecular basis of phenotypic evolution, while at the same time informing more accurate models of regulatory network organization.

Understanding network evolution is an essential step in connecting our knowledge of molecular evolution to the observed phenotypic diversity, yet it remains relatively poorly characterized in vertebrates. While microorganisms have been studied extensively, large-genome organisms with smaller effective population sizes may follow different evolutionary rules. In mammals, many cross-species comparisons have focused on gene dosage changes between evolutionarily distant species such as humans and mice [3, 4]. However, given the architecture of mammalian genomes, changes in transcription factor activity (trans effects) and modifications to cis-regulatory elements (cis effects) are likely to represent more immediate and widespread sources of regulatory evolution than dosage alone. To study these processes, it is advantageous to focus on module divergence among closely related species.

When discussing module conservation, it is important to distinguish between two conceptually different aspects. One is the conservation of a module’s overall expression pattern (often summarized by its “eigengene”), which reflects the combined activity of the genes in the module [5–7]. The other aspect is the conservation of the module’s network topology, defined by the pattern and strength of regulatory relationships among its member genes [8, 9]. These two aspects can diverge: a module may retain a similar expression profile across species even while its internal regulatory wiring changes. Such topological changes can be compensatory, maintaining similar functional outputs despite underlying mechanistic rewiring, a phenomenon called developmental system drift [10]. In the present work, we focus on conservation and divergence in terms of module topology.

The emergence of single-cell RNA sequencing (scRNA-seq) technologies has made it possible to study GRNs at an unprecedented scale and resolution. Profiling thousands to millions of individual cells in a single experiment provides sufficient observations for the detection of co-expression patterns [11]. Moreover, analyzing single cells instead of bulk cell-type mixtures is bound to provide more meaningful networks [12, 13]. However, measures of expression similarity are still affected by substantial biological and technical noise, including cellular stress, dropouts, background contamination, and variation in sequencing depth [14–16].

This low signal-to-noise ratio is especially challenging in a cross-species setting. A common strategy for comparing GRNs across species is to define modules independently in each species and quantify their overlap. However, this approach is highly sensitive to false positives and false negatives in module detection. Even if the true underlying modules are identical, limited power (e.g., $\sim$50% in each species) already reduces the observed overlap to $\sim$33%. False positives further reduce the perceived conservation, as they rarely overlap, especially for small modules. Because error rates also depend on gene expression levels [14–16], highly expressed regulators may appear more conserved. A fair evolutionary comparison, therefore, requires (1) more robust statistical measures than membership overlap, and (2) an explicit adjustment for the sources of variance that underlie differences in power.

Our software, Cross-species Comparison of Networks (CroCoNet, https://hellmann-lab.github.io/CroCoNet/), is designed to meet both of these requirements. To address the first, we incorporate module preservation statistics to compare module topologies across species. To address the second, we use variation observed across biological replicates to correct the preservation scores for confounding factors.

## Results

### Overview of the CroCoNet pipeline

**Fig. 1:**
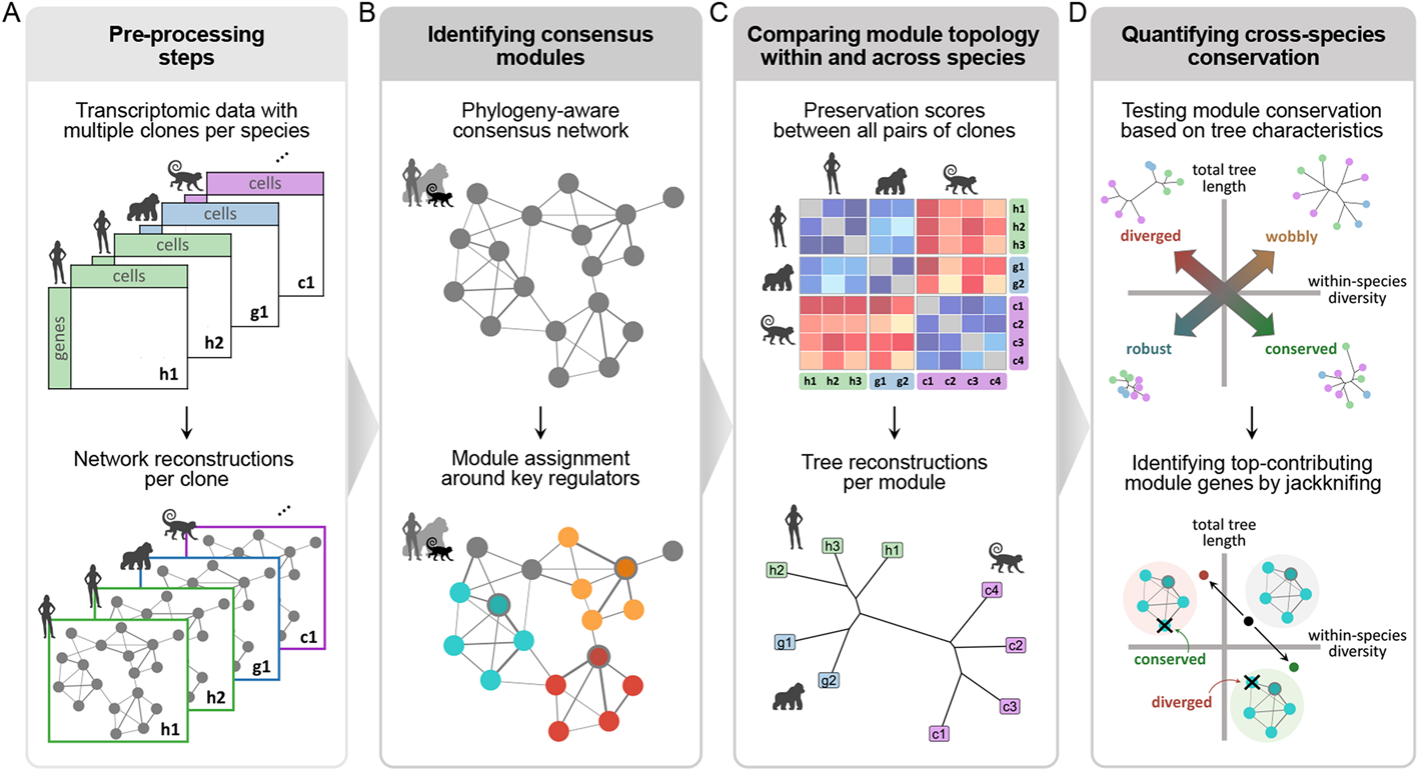
Overview of the CroCoNet pipeline. **A** The input for the pipeline is one network reconstruction per biological replicate. **B** The networks are combined into a single consensus network across replicates and species. Co-expression modules of chosen key regulators are assigned based on the consensus network. **C** The modules are compared between all replicates and species using a preservation score [8]. These scores are converted into a distance matrix, which is then used to reconstruct a neighbor-joining tree. **D** Contrasting the total tree length with the sum of all within-species branch lengths provides a noise-corrected divergence measure. Jackknifing the modules provides a metric for robustness and also allows us to identify influential target genes that contribute the most to the module conservation or divergence

CroCoNet consists of three main steps: 1) identifying consensus modules by integrating information from all species, 2) comparing module topology across species as well as across replicates of the same species, and 3) quantifying module divergence by contrasting the degree of topological differences within versus across species (Fig. 1).

CroCoNet is designed for the analysis of scRNA-seq data from multiple species with multiple biological replicates per species. The experimental conditions, pseudotime trajectory, and cell type composition should be as comparable across species as possible to ensure meaningful results. In addition, the analyzed gene set needs to be matched across species to yield functionally equivalent features that form the basis of network analysis.

The input for CroCoNet is a set of networks, one for each biological replicate (Fig. 1A). Network inference must therefore be performed prior to running CroCoNet, using any method of choice that produces adjacency measures. In our examples, we used two methods: GRNBoost2 [17], a gradient boosting-based approach that performed well in multiple benchmarks [15, 18], and Spearman’s correlation, a simple and computationally efficient alternative. Users must also provide a list of putative regulators, which can be defined based on prior knowledge, data-driven criteria, or a combination of the two. In our examples, we used transcriptional regulators (TRs), i.e. transcription factors (TFs) or chromatin remodelers with a DNA-binding domain and a known binding motif [19, 20] that also showed above-noise expression variance in at least one species of our dataset.

The CroCoNet pipeline begins with the identification of consensus co-expression modules (Fig. 1B). To this end, replicate-wise networks are first combined into a consensus network. Consensus modules are then assigned around the provided regulators based on regulator–target adjacencies (i.e., how strongly a gene is connected to the central regulator) and intramodular connectivities (i.e., how strongly a gene is connected to the rest of the module). Our module assignment uses a dynamic filtering procedure in which modules must stay above a minimum gene count but are otherwise free to vary in size.

The second step is to compare each module between all pairs of replicates, both within and across species (Fig. 1C). To this end, we adapted two preservation statistics suggested by Langfelder et al.: the correlation of adjacencies and the correlation of intramodular connectivities [8]. Both are designed to quantify the degree of topological similarity between two networks but strike a different balance between sensitivity and specificity. These pairwise preservation statistics are converted into a distance matrix, which is then summarized as a neighbor-joining tree.

In the final step, tree-based statistics are used to derive a metric of module divergence (Fig. 1D). In the spirit of many tests commonly used to infer selection from sequence variation [21, 22], we contrast differences within and between species to estimate the rate of meaningful changes. In the context of network analysis, within-species diversity is shaped not only by genetic differences across individuals but also to a large extent by environmental and technical noise. The sum total of these factors — what we measure here as within-species diversity — can be so large that it might obscure true between-species differences and therefore must be corrected for. Fitting a linear model with the total tree length as the dependent variable and within-species diversity as the independent variable yields a slope that informs us about the average contribution of between-species differences to total network variability. The residuals of the linear regression model reflect deviations from this average contribution and can thus be used as measures of module divergence.

### Applying CroCoNet to primate scRNA-seq data

To demonstrate the utility of CroCoNet, we applied it to two primate cross-species scRNA-seq datasets. The first dataset, which was generated in our lab, profiles the early neural differentiation of human, gorilla, and cynomolgus macaque induced pluripotent stem cells (iPSCs). In addition, we analyzed a published dataset comparing brain samples from five primate species [23, 24] (Additional file 1: Fig. S1). Throughout the manuscript, we focus on the neural differentiation data as the main example and only provide complementary insights from the brain data.

**Fig. 2:**
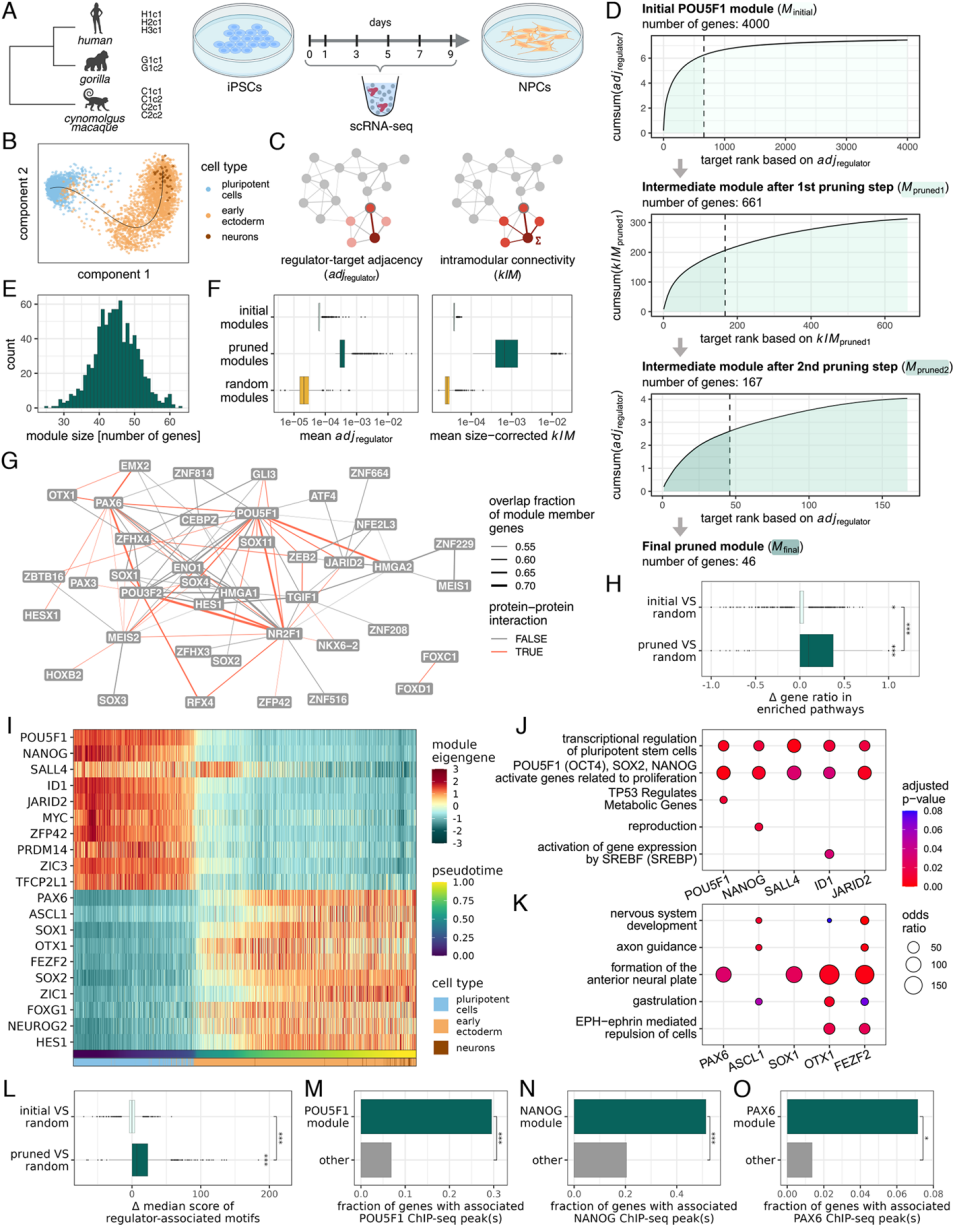
Assigning biologically meaningful consensus modules. **A** Experimental setup of the scRNA-seq experiment. **B** Pseudotime trajectory with cells colored by cell type. **C** Calculation of the regulator–target adjacency ($adj_{\textrm{regulator}}$) and the intramodular connectivity (kIM), the statistics used to prune the modules. **D** Dynamic module pruning, exemplified by the *POU5F1* module. We iteratively calculate the cumulative sum curve of $adj_{\textrm{regulator}}$ or *kIM* and keep targets ranking above the knee point of the curve. **E** Distribution of module sizes after pruning. **F** Mean $adj_{\textrm{regulator}}$ and mean size-corrected *kIM* (module-level summaries of the statistics underlying pruning) of the initial, pruned and random modules. **G** Module overlaps and protein–protein interactions of the regulators. Nodes represent modules associated with the regulators on the label and edge weights represent the overlap fraction of module members. Only the 100 highest overlaps are shown. Red edges indicate that the regulators interact based on STRINGdb [28]. **H** Pathway enrichment was quantified per module as the fraction of genes associated with any enriched Reactome pathway. The plot shows how the enrichment in the initial and pruned modules differs relative to the random modules. **I** Pseudotime trajectories of the module eigengenes for 10 pluripotency [29–34] and 10 early neural markers [35–40]. The eigengene calculation was based on the positively correlated targets. **J**, **K** Pathways with the highest enrichment across the modules of 5 pluripotency markers and 5 neural differentiation markers. **L** Each target gene was scored for binding motifs of the module’s central regulator in the human genome (see Methods). The plot shows how the module-level summaries of these motif scores differ in the initial and pruned modules relative to the random modules. **M**-**O** POU5F1 ChIP-seq peaks [41], NANOG ChIP-seq peaks [42] and PAX6 ChIP-seq peaks [43] are enriched near the member genes of the corresponding modules. **H**, **L**-**O** Asterisks indicate significance: * *p* < 0.05, ** *p* < 0.01, *** *p* < 0.001

To obtain the main example dataset, three iPSC clones from three human individuals, two iPSC clones from one gorilla individual, and four iPSC clones from two cynomolgus macaque individuals (Additional file 2: Table S1) were differentiated into neural progenitor cells (NPCs). During the 9-day differentiation process, cells were profiled using scRNA-seq at six different time points (Fig. 2A). To create a common feature space across all three species, we transferred the human GENCODE gene models [25] to the gorilla and cynomolgus macaque genomes using Liftoff [26]. Note that this approach is only feasible for closely related species and only makes sense when the reference gene models are superior [27]. After the preprocessing steps, pseudotime analysis of the $\sim$4,000 cells revealed a clear trajectory from pluripotent stem cells to early ectoderm and neurons (Fig. 2B) that aligned well across the three species (Additional file 1: Fig. S2A). With comparability across species confirmed, we used GRNBoost2 [17] to reconstruct fully connected GRNs per replicate (Additional file 1: Fig. S2B, see Methods, section “Network inference”).

For the brain data, we used the published count matrices and cell type annotations distinguishing 18 neural and 6 glial subclasses. To ensure an unbiased network inference, we subsampled all replicates to the same number of cells with the same cell type proportions, resulting in $\sim$9,500 cells per replicate (Additional file 2: Table S2). In the case of this larger dataset, we used gene–gene Spearman’s correlations to infer replicate-wise networks, because the runtime of GRNBoost2 was prohibitively slow.

In both cases, these preprocessing steps produced one undirected, unsigned GRN per biological replicate, and these sets of networks served as inputs for the CroCoNet pipeline.

### Consensus network and module assignment

The first step of CroCoNet is to assign consensus modules jointly for all species. The goal is to create modules that are unbiased and meaningful – unbiased in the sense that edges are equally likely to be detected in all species and meaningful in that they are enriched for the actual targets of the regulator.

To achieve this, replicate-wise GRNs were combined into one consensus GRN by calculating the weighted mean of replicate-wise adjacencies per edge. To avoid biasing the consensus network, adjacencies were weighted according to the species’ phylogeny and the number of replicates, so that, in essence, replicates are down-weighted if they belong to 1) closely related species, or 2) species with many replicates. Based on the consensus adjacencies between all network genes and the 836 regulators identified as highly variable in the differentiation dataset (Additional file 1: Fig. S2C-D, see Supplementary Methods, section “Selection of central regulators”), we assigned initial modules, each containing 4,000 genes. These modules were then pruned using a dynamic pruning approach that iteratively selects genes based on two metrics: the regulator–target adjacency and the intramodular connectivity (Fig. 2C). In each pruning step, we calculated the cumulative sum curves based on one of these two metrics per module, then kept the targets ranking above the knee point of the curve (Fig. 2D). We continued this iterative filtering as long as all modules contained at least 20 genes, resulting in a median module size of 45 (Fig. 2E-F, Additional file 2: Tables S3–S4).

Note that, in contrast to WGCNA [44], from which we adapted the preservation statistics, module memberships in CroCoNet are not mutually exclusive. We consider this important, as a realistic representation of gene regulation should also capture the cooperative nature of TRs. Using protein–protein interaction data from STRINGdb [28], we confirmed that interacting regulators in our network tend to share more module member genes than non-interacting ones (Wilcoxon test, $n_1 = 17,670$, $n_2 = 331,360$, $p < 1\cdot 10^{-16}$, Fig. 2G, Additional file 1: Fig. S3A-C). This highlights that CroCoNet effectively captures cooperative regulatory mechanisms.

To assess the biological relevance of the consensus modules, we first examined the enrichment of module genes in known biological pathways. As a reference point for all such module evaluations, we created random modules alongside the actual modules. To this end, we drew target genes at random to match the size of the corresponding pruned module for each regulator (Fig. 2F). The pruned modules contained a significantly higher proportion of genes associated with enriched Reactome pathways [45, 46] compared with the random or initial modules (paired Wilcoxon tests, $n = 836$, $p < 1\cdot 10^{-16}$ in both cases, Fig. 2H, Additional file 1: Fig. S3D). Moreover, as expected for a differentiation trajectory from iPSCs to neural progenitors, the module eigengenes [5] of pluripotency markers were down-regulated toward later pseudotime stages, and their target genes were enriched for pluripotency-related pathways such as *Transcriptional regulation of pluripotent stem cells* (Fig. 2I,J). In contrast, the module eigengenes of early neural markers showed increasing expression over pseudotime, and their target genes were enriched for pathways such as *Formation of the anterior neural plate* (Fig. 2I,K).

Next, we hypothesized that a good module should also be enriched for direct targets of the central regulator. We first tested this hypothesis indirectly by examining the enrichment of the regulator’s binding motifs within putative cis-regulatory elements (CREs) of its target genes (see Methods, section “Inferring binding potential and binding site divergence of the regulators”). We indeed observed the expected enrichment in the pruned modules compared with the random and initial modules (paired Wilcoxon tests, $n = 836$, $p < 1\cdot 10^{-16}$ in both cases, Fig. 2L, Additional file 1: Fig. S3D-E). We then sought more direct evidence by leveraging published ChIP-seq data for POU5F1 [41], NANOG [42], and PAX6 [43] (see Supplementary Methods, section “ChIP-seq enrichment”). Consistent with our hypothesis, the fraction of genes with at least one associated POU5F1, NANOG or PAX6 ChIP-seq peak was significantly higher in the corresponding module than among other network genes (Fisher’s exact test, odds ratio = 5.72 and $p = 5\cdot 10^{-6}$ for POU5F1, odds ratio = 4.11 and $p = 3\cdot 10^{-5}$ for NANOG, and odds ratio = 5.58 and $p = 0.02$ for PAX6, Fig. 2M-O).

To further evaluate our pruning strategy, we compared it to the commonly used strategy of simply selecting the 50 strongest targets of each regulator [6, 47]. We assessed module quality by calculating the phylogenetic signal strength and the difference in preservation between actual and random modules (see Methods, section “Module preservation, tree reconstruction and module filtering”). We found that the dynamic pruning approach outperformed the top-50 strategy in both aspects (Additional file 1: Fig. S4A-H).

Taken together, these results indicate that the CroCoNet consensus modules capture biologically relevant information. In addition, they provide a better balance between false positives and false negatives than fixed-size approaches, leading to a higher signal-to-noise ratio for cross-species comparisons.

### Preservation statistics to compare module topologies

After identifying consensus modules, we quantified how well-preserved module topologies are across replicates and species. We relied on the joint module assignment derived from the consensus network but compared connection patterns directly between the replicate-wise networks. To define suitable metrics, we incorporated two preservation statistics from the WGCNA workflow introduced by Langfelder et al. [8] into CroCoNet. The correlation of adjacencies (*cor.adj*) captures fine-grained topology at the level of individual edges, while the correlation of intramodular connectivities (*cor.kIM*) reflects higher-level topology based on intramodular connectivities — gene-level summaries of the underlying adjacencies (Additional file 1: Fig. S5A).

**Fig. 3:**
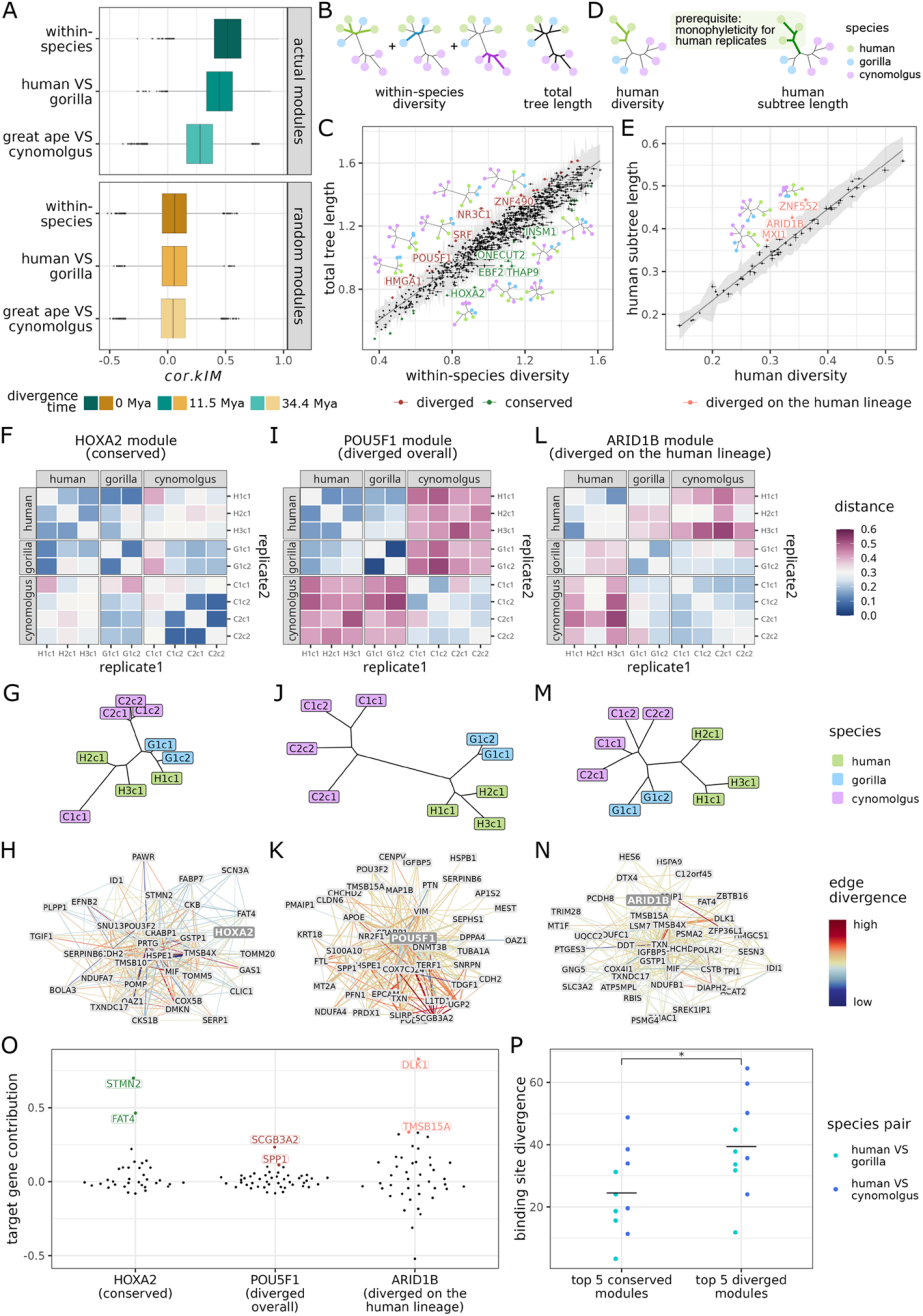
Quantifying the cross-species divergence of network modules. **A** Distribution of *cor*.*kIM* for the actual and corresponding random modules, split by divergence time of the replicates compared. **B** Total tree length and within-species diversity. **C** Quantification of overall module divergence. A linear model was fitted between the total tree lengths and within-species diversities across all modules, and the 95% prediction interval of the regression line (depicted as a gray area) was calculated. Modules that fell above the upper bound or below the lower bound of the prediction interval were considered diverged and conserved, respectively. The five most conserved and five most diverged modules are labeled and displayed using the tree representations. **D** Human diversity and human subtree length. The human subtree length is only defined if the tree is robustly monophyletic for the human replicates. **E** Quantification of module divergence on the human lineage. A linear model was fitted between the human subtree lengths and human diversities across all human-monophyletic modules, and the 95% prediction interval of the regression line (shaded in gray) was calculated. Modules that fell above the upper bound of the prediction interval were considered diverged (labeled and displayed using the tree representations). **F**, **I**, **L** Distance matrices based on *cor.kIM* for the modules *HOXA2* (conserved), *POU5F1* (diverged overall) and *ARID1B* (diverged on the human lineage). **G**, **J**, **M** Neighbor-joining trees for the *HOXA2*, *POU5F1* and *ARID1B* modules. **H**, **K**, **N** The 200 strongest connections of the *HOXA2*, *POU5F1* and *ARID1B* modules. The edge thickness represents the consensus edge weights (scaled per module) and the edge color represents how different the mean edge weights are across the three species ($-\text {log}_{10}F$). **O** Target gene contributions for the *HOXA2*, *POU5F1* and *ARID1B* modules calculated based on jackknifing. For conserved modules, top-contributing targets drive the conservation signal, whereas for diverged modules, they drive the divergence signal. **P** Binding site divergence of the regulators associated with the five most conserved and five most diverged modules

To assess the information content of these metrics, we compared their values for actual versus random modules and to assess the suitability of these metrics for cross-species scRNA-seq analysis, we examined the strength of the phylogenetic signal. Both statistics were higher for the actual modules compared with the random modules, indicating that the modules are informative (Fig. 3A; Additional file 1: Fig. S5B). Moreover, the average preservation followed the expected pattern across different phylogenetic distances: within-species comparisons yielded the highest scores, followed by human–gorilla comparisons, and then great ape (human & gorilla) versus cynomolgus macaque comparisons (Fig. 3A; Additional file 1: Fig. S5B).

Although both metrics appeared suitable by this criterion, *cor.kIM* captured the expected phylogenetic relationships better than *cor.adj* for the neural differentiation dataset (Additional file 1: Fig. S5C–G). In contrast, in the brain dataset, *cor.adj* performed better in terms of retaining phylogenetic information (Additional file 1: Fig. S1E–J). This likely reflects differences in signal-to-noise ratio, which could be influenced by multiple factors, including the GRN inference method (Additional file 1: Fig. S6) and dataset characteristics such as the number of cells and the degree of separation between cell clusters. The differentiation data contain relatively few cells along a continuous developmental trajectory, favoring the use of the robust *cor*.*kIM*, whereas the brain data comprise roughly twenty times more cells from distinct, fully differentiated cell types, favoring the more fine-grained *cor*.*adj*. Because the signal-to-noise ratio of a dataset is generally unknown, we recommend using the functions provided in CroCoNet to identify the optimal preservation metric.

### Quantifying module divergence

It is important to recognize that standard module preservation metrics reflect all sources of variance — not only evolutionary divergence but also technical and environmental noise, which may differ substantially across modules. Thus, to meaningfully interpret preservation metrics as proxies for evolutionary conservation, we must first account for the contributions of technical and environmental variation (Fig. 3B-E).

To accomplish this, we first summarize the preservation scores as neighbor-joining trees (Fig. 1C). The tips of these trees represent the replicates of different species, and the branch lengths are proportional to differences in module topology between the replicates. Like the preservation scores themselves, these branch lengths are affected by biological and technical confounding factors. To establish a baseline for these confounding factors, we use the within-species diversity, which we measure as the total length of all within-species subtrees (Fig. 3B). We capture the expected contribution of the confounding factors to the total module variability by fitting a linear regression model between the total tree length and the within-species diversity across all modules (Fig. 3C). Modules in the lower-left corner yield short trees, suggesting robustness to technical and environmental noise, while modules in the upper-right corner produce trees with long branches and little phylogenetic signal (Fig. 1D). Indeed, random modules also fall into the upper-right corner, supporting the interpretation that these trees are dominated by noise. We therefore exclude modules that fall within the distribution of random modules (Additional file 1: Fig. S4F).

Finally, the residuals from the regression line reflect the variance of the total tree length that cannot be explained by within-species diversity. In biological terms, this component of variance captures the contribution of between-species differences to the overall topological variability of a module. Positive residuals correspond to a greater-than-expected contribution of between-species differences to total tree length, and negative residuals correspond to lower-than-expected contributions. To identify modules with strong deviations from the overall trend, we calculated the 95% prediction interval of the linear fit and considered modules outside this prediction interval as conserved or diverged (Fig. 3C, Additional file 2: Tables S5-S6, see Methods, section “Quantification of cross-species divergence”).

**Fig. 4:**
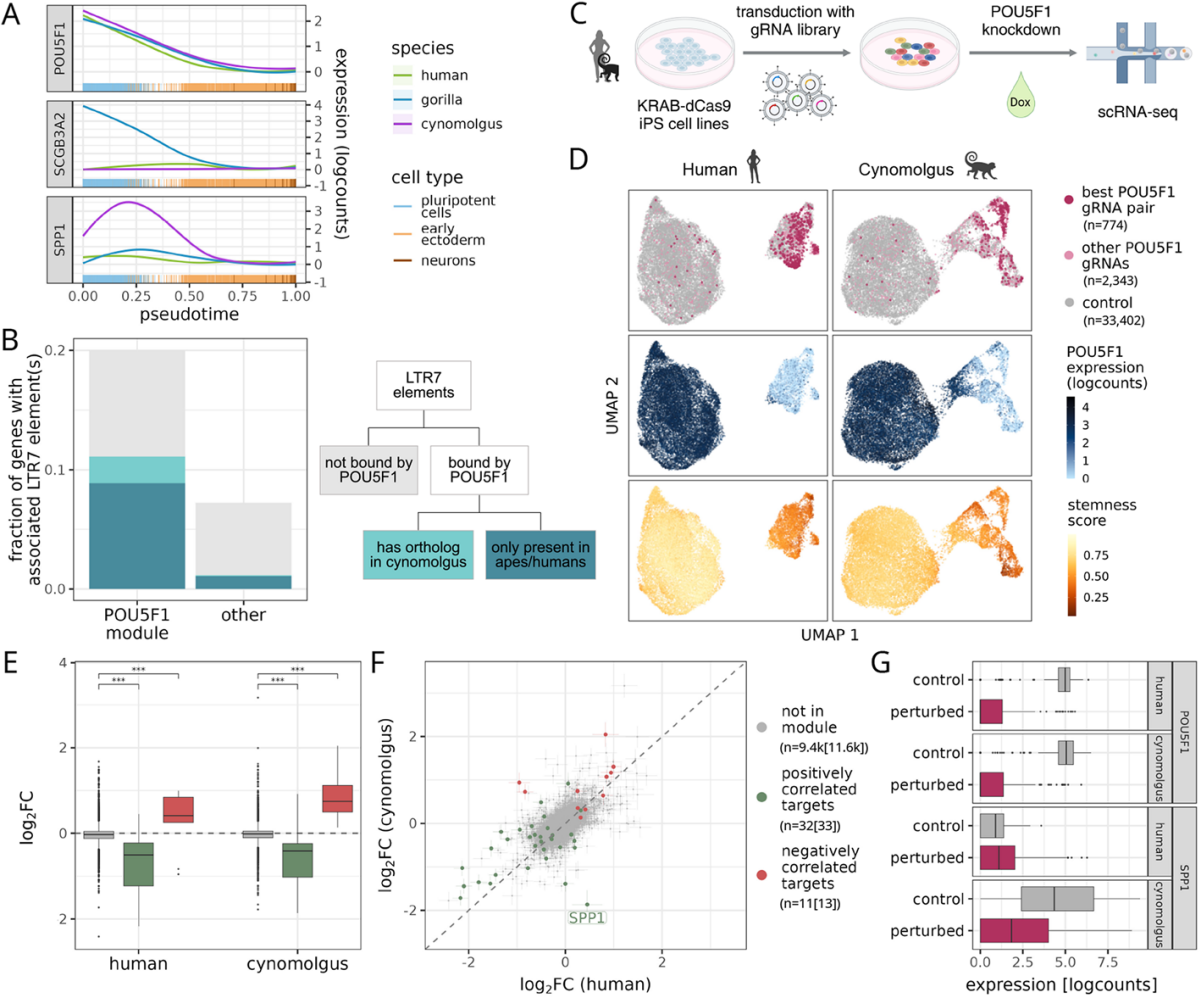
Additional evidence for the divergence of the *POU5F1* module. **A** Expression profiles of *POU5F1* and its two most diverged targets, *SCGB3A2* and *SPP1*. **B** LTR7 enrichment near *POU5F1* module member genes. Compared with all other network genes, in the vicinity of the *POU5F1* module members we detected an enrichment of LTR7 elements (Fisher’s exact test, odds ratio = 3.20, $p = 0.005$), POU5F1-bound LTR7 elements (Fisher’s exact test, odds ratio = 10.6, $p = 0.0002$) and POU5F1-bound LTR7 elements that are only present in great apes or humans (Fisher’s exact test, odds ratio = 8.99, $p = 0.001$). **C** Experimental setup of the single-cell CRISPRi screen. Two human and two cynomolgus macaque iPSC lines with inducible dCas9-KRAB constructs were transduced with species-specific gRNA libraries containing *POU5F1*-targeting and control gRNAs. Five days after dCas9 induction with doxycycline, the gRNAs and transcriptomes were profiled using scRNA-seq. **D** UMAP representation of the *POU5F1*-perturbed and control cells. From top to bottom, cells are colored by gRNA identity, *POU5F1* expression in log-normalized counts, and stemness score. **E** Log fold changes of positively correlated (in green) and negatively correlated (in red) CroCoNet *POU5F1* module member genes as well as non-module genes (in gray) upon *POU5F1* perturbation in human and cynomolgus. Asterisks indicate significance: * *p* < 0.05, ** *p* < 0.01, *** *p* < 0.001. **F** Log fold changes upon *POU5F1* perturbation in human and cynomolgus. Gene counts of positively and negatively correlated *POU5F1* module member genes and non-module genes are indicated below the legend elements, with the number of genes also expressed in the CRISPRi screen shown in brackets. *SPP1*, the most differentially regulated gene in the CRISPRi screen, is labeled. **G** Expression of *POU5F1* and *SPP1* in *POU5F1*-perturbed and control cells in human and cynomolgus macaque

For the differentiation dataset, we identified 20 conserved and 24 diverged modules (Additional file 1: Fig. S7A-F). Among the most conserved modules, we found several expected regulators. For example, *HOXA2* (Fig. 3F-H), like many other Hox genes, shows strong conservation in its sequence, regulatory landscape, and function across vertebrates [48–50]. In contrast, the most diverged module is associated with *NR3C1*, the gene encoding the glucocorticoid receptor, which has a largely conserved protein sequence as well [51], but shows notable differences in hippocampal expression between humans and rhesus macaques [52]. Interestingly, the module with the most robust divergence signal, *POU5F1* (*OCT4*), is a pluripotency factor that is deemed to have high functional and sequence conservation [51, 53], and shows a very conserved expression pattern in our data (Figs. 3I-K, 4A).

To identify influential genes within diverged and conserved modules, we developed a metric to assess the contribution of each target gene to the overall module divergence score based on jackknifing ($c_i$, Additional file 1: Fig. S8, see Methods, section “Target gene contribution”). A positive score indicates that the presence of a target gene strengthens the signal of conservation or divergence, while a negative score means that the target gene weakens the signal. If $c_i \ge 1$, the categorization of the module as diverged or conserved was solely dependent on one gene. This was the case only for one module centered on the regulator *PNRC2*; for most modules, multiple genes contribute to the module conservation status (Fig. 3O, Additional file 1: Fig. S9, Additional file 2: Table S7). Most importantly, this metric should direct users to regulator–target interactions that are most promising for further investigation.

### Lineage-specific divergence

The approach described above for identifying conserved and diverged modules provides a measure of overall divergence across the entire tree. However, because the longest lineages have the strongest influence, this metric is dominated by the divergence between cynomolgus macaques and great apes in the neural differentiation dataset.

In many cases, however, the primary interest lies in lineage-specific selection, for which one would need to identify accelerated evolution along the lineage of interest. To address this, we need a well-defined lineage; for example, we define the human lineage as the branch leading from the most recent common ancestor (MRCA) of humans and gorillas to the human MRCA, using the macaque as an outgroup (Fig. 3D). A minimal requirement for this analysis is that the human replicates form a monophyletic group. If the lineage is indeed well-defined, we quantify module divergence by contrasting the length of the human subtree, including the human lineage, to the human diversity (Fig. 3D). This allows us to assess module divergence specifically on the human lineage (Fig. 3E; Additional file 2: Table S5).

Following this approach, we identified three regulators with diverged modules on the human lineage (Additional file 1: Fig. S7G-I), including *ARID1B*, which encodes a chromatin remodeler with reduced expression in the human brain relative to the brains of other primates [54, 55] (Fig. 3L-N). Analogous to this human-centered analysis, an analysis focusing on the gorilla lineage identified 11 diverged modules, with *CEBPB* as the most diverged one (Additional file 1: Fig. S10, Additional file 2: Table S5).

### Interpreting topological divergence: cis versus trans contributions

Ultimately, a module’s divergence is determined by the sum total of cis- and trans-regulatory effects. On the one hand, if trans effects are the primary drivers of module divergence, we expect the regulators of diverged modules to have either more diverged expression patterns or more diverged protein-coding sequences than the regulators of conserved modules. Indeed, for certain diverged modules such as *NR3C1*, the expression profiles of the regulators themselves differ markedly between species (Additional file 1: Fig. S9D). Overall, however, we do not find a significant difference in protein sequence or expression pattern divergence between the regulators of conserved and diverged modules (Additional file 1: Fig. S11A-D; Additional file 2: Table S5).

On the other hand, if the rewiring of a regulatory network occurs via changes in the cis-regulatory landscape, we expect to see a high divergence in the binding sites of the regulator within CREs associated with its target genes. To obtain an estimate of binding site divergence, we defined putative CREs per species as iPSC- and NPC-associated ATAC-seq peaks within 20 kb of each gene’s transcription start site (TSS), and then scored the regulator’s overall binding potential across these CREs for each target gene and species (see Methods, section “Inferring binding potential and binding site divergence of the regulators”). In this way, we also scored non-orthologous CREs and TF binding sites as potentially equivalent regulators. Although such sequence-based binding predictions are far from reliable at the gene level, summarizing them across modules shows that the five most diverged modules indeed have higher binding site divergence than the five most conserved modules (Fig. 3P, Additional file 1: Fig. S11E-F, Additional file 2: Table S5). Even though there is no overall correlation between binding site and network divergence, this top-five comparison still suggests that cis-regulatory changes can be important drivers of module divergence.

That said, cis- and trans-changes of a network are by no means mutually exclusive; both clearly contribute to network rewiring. It is noteworthy that CroCoNet – despite being based solely on co-expression – can detect module divergence arising from both cis- and trans-acting changes.

### Rewiring of the *POU5F1* module

The second most diverged module, *POU5F1*, is a potential example of cis-regulatory rewiring: the protein sequence is relatively conserved between human and cynomolgus macaque (99% amino acid identity) and 100% conserved between human and gorilla. Additionally, there is no evidence of expression pattern divergence for *POU5F1* itself (Fig. 4A). Moreover, based on our jackknife analysis, the rewiring does not hinge on a single target gene; instead, cis-regulatory changes must have affected many module members (Additional file 1: Fig. S8B, Fig. 3O). Regulatory changes are particularly evident when inspecting the expression trajectories of *SCGB3A2* and *SPP1*, the two target genes that contribute the most to module divergence (Fig. 4A).

One proposed mechanism for network rewiring is the exaptation of transposable elements (TEs) [56]. The *POU5F1* network might have been affected by recent insertions of HERVH elements in particular. They have the capacity to reorganize the 3D genome structure in iPSCs [57] and their terminal repeats (LTR7) carry binding sites for multiple pluripotency factors, including POU5F1 [58–60]. Indeed, the *SCGB3A2* isoform reported to be expressed in human iPSCs is a fusion transcript with a great ape-specific HERVH insertion and is only expressed in more naive-like subpopulations [59]. Although among our iPSC lines *SCGB3A2* is only expressed in gorilla, the ATAC-seq data show that the LTR7 elements are also active in humans, suggesting that *SCGB3A2* is poised for expression, whereas this is not observed in the macaque (Additional file 1: Fig. S12). For *SPP1*, the LTR7 involvement is less direct: an HERVH insertion likely altered the 3D chromatin structure and disrupted the interaction between the *SPP1* promoter and a distal POU5F1-binding CRE [61] (Additional file 1: Fig. S13-S14, see Supplementary Methods, section “3D genome architecture upstream of *SPP1*”).

To assess whether this extends beyond these two genes, we investigated the presence of LTR7 elements near the promoters of all *POU5F1* module member genes in the human genome (Fig. 4B). Compared with all other genes expressed in iPSCs, we detected a significant enrichment of LTR7 elements (Fisher’s exact test, odds ratio = 3.20, $p = 0.005$), and an even stronger enrichment of LTR7 elements that are bound by POU5F1 in human embryonic stem cells based on ChIP-seq data (Fisher’s exact test, odds ratio = 10.6, $p = 0.0002$) [62]. Furthermore, many of the POU5F1-bound LTR7 elements near *POU5F1* module member genes are great ape- or human-specific, including two LTR7 elements in the vicinity of *SPP1* and three in the vicinity of *SCGB3A2*. Therefore, we hypothesize that these recent HERVH insertions contributed to the rewiring of the *POU5F1* module on the great ape lineage.

### Validation of the *POU5F1* module divergence by CRISPRi

To validate the CroCoNet approach for assigning co-expression modules and assessing their divergence, we perturbed *POU5F1* in two human and two cynomolgus iPSC lines using CRISPR interference (CRISPRi) (Fig. 4C, Additional file 2: Tables S1, S8-S9). CRISPRi perturbations enable the identification of target genes for a given regulator through a simple differential expression analysis between perturbed and unperturbed cells. When performed across species, differential expression comparisons provide a measure of regulatory divergence by identifying differentially regulated genes, i.e., genes whose responses to the knockdown differ between species (see Methods, section “*POU5F1* perturbations using CRISPRi”).

Using scRNA-seq, we confirmed the efficiency of the perturbation: cells expressing a *POU5F1*-targeting gRNA showed reduced *POU5F1* expression and a marked decrease in the stemness index, indicating successful repression of *POU5F1* (Fig. 4D). Of the 46 genes in the *POU5F1* CroCoNet module, 43 were detected as expressed in the CRISPRi dataset and were therefore available for assessing module membership. The three excluded genes are expressed either only in gorilla iPSCs (*SCGB3A2*) or only in NPCs (*NR2F1* and *POU3F2*), and could thus not be detected in the CRISPRi cell lines. Strikingly, all 43 expressed module genes were identified as *POU5F1* targets in the CRISPRi differential expression analysis, providing strong validation of the CroCoNet-derived consensus module (Fisher’s exact test, odds ratio = Inf, $p = 2\cdot 10^{-12}$). Furthermore, the direction of regulation was highly consistent between the CroCoNet inference and the knockdown experiment: genes inferred to be repressed by *POU5F1* were up-regulated upon knockdown, and genes inferred to be activated were down-regulated. In contrast, genes outside the module showed only minimal expression changes (Fig. 4E, Additional file 2: Table S10).

We also found that 28 of the 43 expressed module genes were significantly differentially regulated between the two species. This represents a significant enrichment compared with all other differentially expressed genes (Fisher’s exact test, odds ratio = 4.29, $p = 3\cdot 10^{-6}$), confirming that the *POU5F1* module is indeed diverged between human and cynomolgus iPSCs. The most strongly differentially regulated gene was *SPP1* (Fig. 4F; $\log _2\textrm{FC} = -2.3$, $p_{\textrm{adj}} < 1\cdot 10^{-16}$), which was also identified as the second most diverged *POU5F1* target in the CroCoNet analysis (the most diverged, *SCGB3A2*, could not be tested here; Fig. 3O). Consistent with the expression patterns observed in the neuronal differentiation dataset, *SPP1* showed higher expression in unperturbed cynomolgus iPSCs than in unperturbed human iPSCs (Fig. 4G). Upon *POU5F1* knockdown, *SPP1* was slightly but significantly up-regulated in human cells ($\log _2\textrm{FC} = 0.45$, $p_{\textrm{adj}} = 0.02$), whereas it was significantly down-regulated in cynomolgus cells ($\log _2\textrm{FC} = -1.9$, $p_{\textrm{adj}} < 1\cdot 10^{-16}$). This independently confirms that *SPP1* is regulated differently by *POU5F1* in humans and cynomolgus macaques.

Taken together, the CRISPRi perturbation experiment validates both the module membership and the regulatory divergence of the *POU5F1* module across primates. This example demonstrates that CroCoNet recovers biologically meaningful cross-species differences in network wiring.

## Discussion

CroCoNet, the framework we introduce, is designed to analyze GRN divergence across species, particularly when cis-regulatory rewiring is the driving force.

A prerequisite for such network analyses is a matched feature space across species. In this manuscript, we focus on comparisons across primates, where it is straightforward to define this common feature space based on sequence similarity and synteny. For more distantly related species, previous work has proposed network alignments to identify functionally equivalent genes across species [63]. Moreover, recent advances in large language models have made it increasingly feasible to infer functional homology directly from amino acid sequences, which could further facilitate the construction of shared feature spaces [64, 65]. This still leaves the issue of species-specific genes with no functional equivalents, which is more likely to impact distantly related species. In principle, it is possible to incorporate these genes into the input networks of CroCoNet. They should have nonzero edge weights in only one species and thus be detected as diverged.

The biggest challenge for evolutionary comparisons of GRN architectures is the low power to detect true regulatory connections. This is particularly problematic for single-cell data from closely related species, where a low signal-to-noise ratio is expected. The issue has already been pointed out by Suresh et al. [24] and prompted them to rely not only on their large single-cell brain dataset, but to integrate bulk data, and to only focus on evolutionary network changes that are accompanied by expression profile changes. Other strategies to boost the reliability of the inferred edges involve the integration of complementary data such as TF binding. This approach is implemented in the popular network inference tools SCENIC and SCENIC+ [6, 66]. SCENIC uses sequence information alone to score TF binding in large windows around the TSS, whereas SCENIC+ requires additional scATAC-seq data, which improves the performance substantially. SCORPION takes another route by integrating information from protein–protein interactions [67]. Note that even though comparative approaches could also benefit from multimodal data integration, such data are still rare for non-model organisms. Therefore, methods that are solely based on co-expression networks remain important for cross-species GRN comparisons.

Due to power limitations, previous work has primarily focused on identifying conserved modules, while diverged modules have remained difficult to interpret [3]. For example, in Aibar et al. [6], module conservation was assessed in two ways: conservation of module memberships was evaluated by cross-tabulation, and conservation of module expression patterns was evaluated using AUCell-derived module activities. This analysis suggested that the activity of a module is typically more conserved than the activity of its individual genes. Similarly, in Feregrino et al. [68], who also applied WGCNA preservation scores to single-cell data, the emphasis was on the overall stability of developmental modules across large phylogenetic distances. The main reason these approaches focus on conservation is that, unlike CroCoNet, they do not explicitly account for varying signal-to-noise ratios across modules. The only approach with a similar rationale is the WGCNA-based comparison of GRNs in bulk RNA-seq data from human and chimpanzee brains by Oldham et al. [69]. They identified human-specific regulatory links by contrasting edges inferred from human samples alone with the cross-species average.

With CroCoNet, we provide a quantitative framework to compare GRN modules across species, allowing us to distinguish evolutionary rewiring from technical and environmental noise – an issue that is of particular importance for closely related species. Rather than focusing solely on conservation, CroCoNet emphasizes differences in network topology and uses biological replicates to account for any confounding within-species variability. To our knowledge, there is no other method that provides a metric comparable to our divergence scores. To calculate them, we require properly processed input networks from which we 1) infer co-expression modules, 2) calculate preservation statistics, and 3) quantify meaningful cross-species differences while correcting for unwanted variability.

Although CroCoNet introduces several safeguards to handle noisy data, careful preprocessing and an appropriate experimental design are important. Aside from following general best practices to clean scRNA-seq data [70], CroCoNet requires cross-species comparability. In particular, an approximately even cell number and a similar representation of cell types need to be ensured across species and replicates. While unbiased cell type compositions are crucial for an unbiased cross-species comparison, CroCoNet should be fairly robust to batch effects, because the raw edge weights are always measured within replicates. Batches are only expected to impact the results if they have a strong, isolated effect on a relatively small subset of genes. Besides comparability, the dataset should contain sufficient biological variation. If there is too little signal, CroCoNet will return few or no modules, and the identified modules might not show significant departures from the average conservation level. To help evaluate how informative a module is, CroCoNet offers checkpoints that make use of random modules and phylogenetic information.

The first step of the actual CroCoNet pipeline is to infer co-expression modules. Here, our dynamic pruning approach is designed to strike the right balance between sensitivity and specificity when defining module membership. This is crucial in a comparative setting, because overly inclusive modules risk accumulating false positives, which in turn weakens any signal of conservation. The resulting modules are the units for which we infer our divergence metrics. CroCoNet interprets topological differences through a framework analogous to the McDonald-Kreitman test [21], treating within-species diversity as a nuisance parameter to isolate true phylogenetic signal. Assuming a baseline contribution of technical and environmental noise to total network variability, we rank modules based on their deviation from this expectation; positive residuals indicate accelerated evolution, while negative residuals denote high conservation. While these scores are necessarily relative due to the lack of an absolute neutral reference, this logic — successfully established in the study of gene expression evolution [71–74] — provides a robust means to identify meaningful regulatory rewiring based on noisy single-cell data.

Having defined the statistical framework for quantifying module divergence, we next consider how these signals can be interpreted in terms of regulatory changes. So far, we tested CroCoNet only on undirected networks, as regulatory directionality is difficult to infer reliably from co-expression data alone. However, directionality is implicitly introduced by centering each module on a putative regulator, such that edges are interpreted primarily as reflecting regulatory influence of that regulator on module genes.

CroCoNet is primarily designed to detect diverged modules where disproportionately many edges among module genes have changed between species. Such changes in edge weights can be caused by both cis and trans effects. If the putative regulator of a module has a diverged expression profile, the most parsimonious interpretation of a diverged network would be via trans effects. Note, that differences in protein sequence or expression, that do not lead to different regulator-gene interactions, will not be detected by our network divergence metric. For example, if a regulator shows a consistent increase in activity across cell types and states, which also leads to a consistent increase in target gene activities, this would not amount to a topological difference. Such changes in module activity could only be detected by comparing the module eigengenes, which is implemented as a complementary analysis in CroCoNet.

While trans effects might already be obvious from a differential expression analysis of the regulators alone, disentangling cis effects based on transcriptomics data is a non-trivial task. A unique feature of CroCoNet is that it can detect divergence originating from cis effects. This feature makes CroCoNet particularly well suited to study network rewiring across closely related species, where cis-regulatory evolution is thought to play a major role.

CroCoNet also provides tools to investigate the most conserved and diverged modules in greater depth (Figs. 3O, 4A). These tools are particularly useful for prioritizing target genes and distinguishing cis from trans effects. Notably, *POU5F1* (*OCT4*), the central regulator of the second most diverged module, shows a highly conserved expression trajectory (Fig. 4A) and likely represents an example of cis-regulatory rewiring.

Kunarso et al. [58] showed that although POU5F1 is a core pluripotency factor in both humans and mice, its genomic binding locations are largely non-orthologous, with transposable elements contributing roughly one quarter of the binding sites in each species. Yet, only a subset of these TE-derived “proto-enhancers” exerts detectable regulatory effects [75, 76], leaving open the question of whether such cis-regulatory turnover meaningfully reshapes the gene regulatory network. Here, we show that this turnover indeed corresponds to measurable changes in the topology of the *POU5F1* module. CroCoNet is well suited to such analyses because it directly compares GRN topologies across species, allowing us to assess the extent to which cis-regulatory rewiring translates into network-level rewiring. What remains unclear is whether these network differences reflect adaptive divergence in early development, or instead represent developmental system drift, where changes in regulatory architecture do not alter core functional outcomes [10].

## Conclusions

In conclusion, CroCoNet provides a phylogeny-aware framework for the quantitative comparison of gene regulatory networks across species. By explicitly modeling within-species variability across biological replicates, our method addresses the low signal-to-noise ratio inherent to single-cell transcriptomics and makes it possible to discern true evolutionary signals from technical and biological noise. Thus, CroCoNet moves beyond standard comparisons focused on conservation alone and captures regulatory rewiring as well. This allows us to reliably identify both conserved and diverged network modules in transcriptomics data.

In the future, the utility of CroCoNet could benefit from advances in preprocessing and network inference methods. In particular, AI-based approaches may improve the input networks by providing a better common feature space, more efficient data denoising, and more accurate machine-learning-based edge weights. CroCoNet could also be extended to incorporate additional modalities, particularly ATAC-seq data in conjunction with new AI-based motif-finding methods (e.g. [77]). Beyond co-expression GRNs, the principles underlying CroCoNet could also be applied in the context of other biological networks, including comparative analyses of metabolic or protein–protein interaction networks.

## Methods

### Cell lines

For the scRNA-seq, ATAC-seq, long-read RNA-seq and CRISPRi experiments in this study, we used a total of 17 human (*Homo sapiens*), gorilla (*Gorilla gorilla*) and cynomolgus macaque (*Macaca fascicularis*) iPSC lines (Additional file 2: Table S1). All lines have been published previously [78–82], except for the human cell line 30B2 and the cynomolgus macaque cell line 46B6. The iPSC lines were reprogrammed from urine-derived stem cells, peripheral blood mononuclear cells (PBMCs), or dermal fibroblasts using integration-free Sendai virus-based OSKM vectors. All cell lines were characterized, validated and authenticated according to best practices as described previously [79, 83, 84]. As part of the validations, they tested negative for mycoplasma and were confirmed to be free of the reprogramming vector.

### Experimental procedures and data processing

To generate the neural differentiation dataset, three human, two gorilla and four cynomolgus macaque iPSC lines (Additional file 2: Table S1) were differentiated into NPCs over the course of nine days using dual-SMAD inhibition [85, 86]. On days 0, 1, 3, 5, 7 and 9, cells were sampled and profiled using mcSCRB-seq [87].

Reads were mapped to hg38, gorGor6 and macFas6 using zUMIs [88]. For the human genome, we used the GENCODE gene build (release 32). For the gorilla and cynomolgus macaque, we transferred the human gene build to the corresponding genomes using Liftoff [26]. We removed low-quality cells and unexpected cell types (mainly neural crest), as well as genes with unsuccessful liftoff, low expression, and unwanted gene types (see Supplementary Methods, section “Processing of the differentiation dataset”). We normalized counts using scran [89] and annotated cell types using SingleR [90] with a human embryoid body dataset as the reference [91]. After batch effect removal [92], we inferred pseudotime using SCORPIUS [93].

For the brain dataset profiling the middle temporal gyrus of five primate species [23], we downloaded the filtered count matrices with the attached metadata from https://data.nemoarchive.org/publication_release/Great_Ape_MTG_Analysis/. We only kept 10x data and removed the donor H18.30.001 due to its atypical cell type composition, as well as the donors H200.1023 and C19.32.006 due to their low cell numbers. This left us with four human, six chimpanzee, four gorilla, three rhesus and three marmoset donors (Additional file 1: Fig. S1A-B, Additional file 2: Table S2).

### Network inference

In case of the differentiation dataset, we normalized counts per replicate using randomized quantile residual transformations prior to network inference [94]. Based on these normalized counts, we inferred networks per replicate with GRNBoost2 [17, 95], specifying all genes in the filtered count matrix as potential regulators. We ran the algorithm 10 times for each replicate with 10 different seeds, then averaged edge weights across the 10 runs and 2 possible directions. Edges that reached the detection limit in none or only one of these 20 network versions were assigned a weight of 0. Filling in zeros ensured that, despite GRNBoost2 not providing importance scores for every possible edge, the resulting networks were still fully connected.

In case of the brain dataset, all donors were subsampled to the same number of cells per cell type, resulting in 9,456 cells per donor (Additional file 1: Fig. S1B). We created ten independent subsamples with this cell number and cell type composition per donor and performed ten independent network reconstructions to retain as much information from the data as possible. For each network reconstruction, we calculated Spearman’s correlations between all gene pairs based on the log-normalized expression matrices across all cell types. The network reconstructions of the different subsamples were then averaged to obtain a single network per donor. The correlation coefficients were transformed into unsigned adjacencies by taking their absolute values.

### Consensus network

We integrated the per-replicate networks into a consensus network in a phylogeny-aware manner, building on the concepts of Phylogenetic Generalized Least Squares (PGLS) [96, 97]. Based on primate divergence time estimates [98], we calculated a phylogenetic similarity matrix (*S*) across all replicates:

1 $$\begin{aligned} S_{ij} = 1 - \frac{t_{ij}}{\max _{i, j \in R} t_{ij}} \end{aligned}$$

where *R* is the set of replicates for which the consensus is calculated, and $t_{ij}$ is the divergence time of the two species replicates *i* and *j* belong to.

Using this similarity matrix, the weights of the replicates ($w_i$) were calculated as:

2 $$\begin{aligned} w_i \propto \frac{1}{\sum \nolimits _{j \in R} S_{ij}} \end{aligned}$$

and were normalized to sum to 1. Finally, the consensus adjacency of an edge ($a_{consensus}$) was calculated as the weighted mean of the adjacencies in each replicate ($a_{i}$):

3 $$\begin{aligned} a_{consensus} = \sum \limits _{i \in R} w_ia_i \end{aligned}$$

### Module assignment

As central regulators for the module assignment, we selected genes that had at least one annotated binding motif in the JASPAR [19] or IMAGE [20] databases and had a variance above the noise level in the data based on variance decomposition [89]. Applying these criteria yielded 836 regulators for the differentiation dataset and 499 regulators for the brain dataset.

Modules associated with these regulators were created and pruned based on the consensus network. Specifically, we used two metrics: 1) the regulator–target adjacency ($a_{\textrm{regulator}}$), defined as the adjacency between the regulator and a gene, and 2) the intramodular connectivity (*kIM*), defined as the sum of a gene’s adjacencies to all other genes within the same module [8]:

4 $$\begin{aligned} kIM_i^{(q)} = \sum \limits _{\begin{array}{c} j \in q\\ j \ne i \end{array}} a_{ij}, \end{aligned}$$

where *q* is the set of genes belonging to a module.

We assigned an initial module to each regulator by selecting its top 4,000 target genes based on $a_{\textrm{regulator}}$. Next, we pruned the modules using an iterative two-step process: 1) we calculated the cumulative sum curve based on $a_{\textrm{regulator}}$ per module, identified the knee point of the curve [99], then kept the targets with a higher $a_{\textrm{regulator}}$ than the knee point, and 2) we repeated the knee-point–based selection for *kIM*. Pruning steps 1) and 2) were repeated until the module sizes became as small as possible without any of them falling below 20 (Additional file 2: Tables S3-S4). For the differentiation dataset, pruned module sizes ranged between 25 and 63, with a median of 45, while for the brain dataset, module sizes ranged between 29 and 67, with a median of 51.

Corresponding to each module, we also created a random module of the same size by randomly drawing genes from the network (without replacement).

### Inferring binding potential and binding site divergence of the regulators

We validated the membership and divergence of the modules in the differentiation dataset based on binding site predictions of the central regulators. To this end, ATAC-seq data were collected from two human, two gorilla and three cynomolgus iPSC samples as well as from two human and two cynomolgus NPC samples (Additional file 2: Table S1) using the Omni-ATAC protocol [100]. Reads were mapped to the same reference genomes as for the scRNA-seq data using BWA-MEM2 [101]. Peaks were called jointly for all replicates per cell type and species using genrich [102]. Peaks in gorilla NPCs were inferred by LiftOver [103] of the human NPC peaks to gorGor6.

Active TSSs were identified in human, gorilla, and cynomolgus iPSCs and NPCs using the GENCODE or Liftoff annotations, long-read RNA-seq, and ATAC-seq data (see Supplementary Methods, section “Peak-to-gene associations”). For each cell type and species, ATAC-seq peaks were associated with genes based on their distance to TSSs [81], allowing a maximum distance of 20 kb. We scored the binding motifs of each central regulator [20, 104] in all peaks associated with the genes in their initial, pruned and random modules using Cluster-Buster [105]. After collapsing redundant motif hits, we calculated the binding potential of a regulator per gene and species by summing the motif scores across all associated peaks. For module validation, the binding potential was further summarized per module using the median.

To investigate cross-species divergence in binding potential, we calculated the absolute differences in binding potential between all species pairs per gene and summarized these differences as the median per species pair and module (Additional file 2: Table S5). To test whether regulators with conserved and diverged network modules differ in terms of their binding site divergence, we fitted the following linear model across the human-gorilla and human-cynomolgus comparisons of conserved and diverged CroCoNet candidates:

5 $$\begin{aligned} \text {binding site divergence} \sim \text {network conservation category + phylogenetic distance} \end{aligned}$$

When contrasting all conserved to all diverged modules, we detected no significant difference in binding site divergence ($\beta = 3.48$, $p = 0.47$), but when contrasting the top five modules from each category, we detected a significantly higher binding site divergence for the regulators of diverged modules ($\beta = 14.9$, $p = 0.02$).

### Module preservation, tree reconstruction and module filtering

We quantified module preservation by calculating the preservation statistics *cor.adj* (correlation of adjacencies) and *cor.kIM* (correlation of intramodular connectivities) [8] for each consensus module between all possible pairs of replicates.

We converted the preservation scores of the chosen statistic (*pres*) into distances ($dist=\frac{1-pres}{2}$) to construct a distance matrix and infer a neighbor-joining tree per module [106]. Based on these trees, we calculated overall tree characteristics (the total tree length and within-species diversity, Fig. 3B) as well as lineage-specific tree characteristics (subtree length and diversity of the species of interest, Fig. 3D).

To assess the uncertainty of these metrics, we used jackknifing, meaning that we removed each member gene of a module and recalculated all preservation scores and tree-based statistics. We used the median and its 95% confidence interval across the jackknifed versions to estimate a statistic of interest for the module as a whole.

We used the overall tree characteristics to remove uninformative modules. For each module, we estimated the probability of originating from the distribution of random modules ($p_{\text {random}}$) and from the distribution of actual modules ($p_{\text {actual}}$), using the probability density functions of the two bivariate normal distributions. If

6 $$\begin{aligned} \frac{p_{\text {actual}}}{p_{\text {actual}} + p_{\text {random}}} > 0.95 \end{aligned}$$

was not met, we excluded the module. This led to the removal of 10 modules for the differentiation dataset and 11 modules for the brain dataset.

### Quantification of cross-species divergence

To characterize the overall divergence of the modules, we fitted a weighted linear model with the total tree length ($L_t$) as the dependent variable and the within-species diversity ($L_w$) as the independent variable ($L_t = \beta L_w + c + \varepsilon$). Specifically, for each module we used the median total tree length and median within-species diversity across its jackknifed versions and weighted the data point inversely proportional to the jackknife variance estimate of the total tree length. We regarded the residuals of each module *m* ($\varepsilon _m$) from this linear model as measures of cross-species module divergence. To identify modules that deviate from the average degree of conservation, we calculated the 95% prediction interval of the regression line. We considered a module diverged if it had a higher total tree length than the upper boundary of the prediction interval, while we considered a module conserved if it had a lower total tree length than the lower boundary of the prediction interval (Additional file 2: Tables S5-S6). For the differentiation dataset, we detected 20 conserved and 24 diverged modules (Fig. 3C), while for the brain dataset, we detected 10 conserved and 15 diverged modules (Additional file 1: Fig. S1J).

It is also possible to identify a robust subset of these conserved and diverged modules by calculating externally studentized residuals, converting them to *p*-values, and applying Benjamini-Hochberg false discovery rate (FDR) control. With the cutoff $p_{\text {adj}} \le 0.1$, we identified 0 robustly conserved modules and 2 robustly diverged modules (*POU5F1*, *HMGA1*) in the differentiation dataset, and 2 robustly conserved modules (*ID1*, *OLIG2*) and 3 robustly diverged modules (*POU3F1*, *PRRX1*, *RCOR1*) in the brain dataset.

To estimate the lineage-specific divergence of a module, we required the module tree to be monophyletic for the species of interest across all jackknife versions. Analogous to the analysis of overall divergence, we fitted a weighted linear model, this time using the species-specific subtree length instead of the total tree length and species-specific diversity instead of the overall within-species diversity. Note that due to the filtering for monophyleticity, we lose the most conserved modules and thus cannot interpret modules below the lower boundary of the prediction interval as conserved.

### Target gene contribution

To identify the most conserved and diverged target genes within a module, we used the results of jackknifing. We relied on the linear model that was fit across all modules between the total tree length and within-species diversity (for overall divergence), or between the subtree length and diversity of a species (for lineage-specific divergence). For each jackknifed module version created by removing a gene *i*, we re-calculated the total tree length ($L_{t,i}$) and within-species diversity ($L_{w,i}$). Based on these, we also obtained the deviation from the original regression line ($\varepsilon _i=L_{t,i} - \hat{L}_{t,i}$, Additional file 2: Table S7). Using the residual of the original module ($\varepsilon _m$), we defined the target gene contribution score ($c_i$) as follows:

7 $$\begin{aligned} c_{i} = \frac{\varepsilon _m - \varepsilon _i}{\varepsilon _m} \end{aligned}$$

### *POU5F1* perturbations using CRISPRi

We perturbed *POU5F1* in human and cynomolgus inducible KRAB-dCas9 iPSC lines [82] (Additional file 2: Table S1) as part of three pooled single-cell CRISPRi screens. We designed gRNAs targeting *POU5F1* (Additional file 2: Table S8) as well as non-targeting control gRNAs (Additional file 2: Table S9). Details of the gRNA design, experimental procedures, and initial data processing are described in Edenhofer et al. (2024) [82], in the Supplementary Methods and in our GitHub repository https://github.com/anita-termeg/gRNA-design.

For the validation of the CroCoNet *POU5F1* module in the differentiation dataset, we selected one gRNA per species with high and comparable knockdown efficiencies (human: GACAGAACTCATACGGCGGG, % KD = 83.9%; cynomolgus: GAAGCCAGGTGTCCCGCCAT, % KD = 84.8%). To further ensure comparability, we kept only batches shared across the two gRNAs and downsampled to an equal number of cells per condition and species, resulting in 288 *POU5F1*-perturbed and 288 unperturbed control cells in both human and cynomolgus.

Next, we fitted a linear mixed-effects model using dream [107] with the design:

8 $$\begin{aligned} \text {gene expr.} \sim \text {species + condition + species:condition + (1 | individual) + (1 | batch)} \end{aligned}$$

We tested three contrasts: 1) perturbed vs. control in human cells, 2) perturbed vs. control in cynomolgus cells, and 3) the interaction between species and condition (i.e. evaluating whether the perturbed–control difference is significantly larger in one of the species). We regarded a downstream gene as significantly differentially expressed (DE) within a species or significantly differentially regulated (DR) across the species if the corresponding contrast had an adjusted *p*-value <0.05 (Benjamini-Hochberg FDR correction). Out of the 10,207 genes tested, 3,871 were DE in human, 3,471 were DE in cynomolgus and 1,720 were DR across the two species, while out of the 43 *POU5F1* module member genes tested, 39 were DE in human, 37 were DE in cynomolgus and 28 were DR across the two species (Additional file 2: Table S10).

## Supplementary Information

Additional file 1: Supplementary Figures S1-S14.

Additional file 2: Supplementary Tables S1-S10.

Additional file 3: Supplementary Methods.

## Data Availability

The CroCoNet R-package is available at https://github.com/Hellmann-Lab/CroCoNet [108] under the GPL-3.0 license, with detailed documentation and a step-by-step tutorial at https://hellmann-lab.github.io/CroCoNet/. The code for all analyses in this manuscript is available at https://github.com/Hellmann-Lab/CroCoNet-analyses [109] under the GPL-3.0 license. A stable version of the code and the related data files have been deposited to the linked Zenodo archive [110]. Datasets generated for this study have been deposited in public repositories. The neural differentiation scRNA-seq data have been deposited in ArrayExpress under the accession number E-MTAB-15695 [111]. The ATAC-seq data of the cell lines G1c2, G1c3 and C1c3 have been deposited in ArrayExpress under accession number E-MTAB-15654 [112]. The long-read RNA-seq data have been deposited in GEO under the accession number GSE326106 [113]. The single-cell CRISPRi data, profiling the effects of the POU5F1 perturbation, have been deposited in GEO under the accession number GSE325993 [114]. All other datasets were previously published and reused for this study. The raw data for the primate brain snRNA-seq dataset are available at the Neuroscience Multi-omic Data Archive (NeMO) under the ID nemo:dat-net1412 [115] and the corresponding processed data can be downloaded from the NeMO Archive repository ”Great Ape MTG Analysis”. The ATAC-seq data of the cell lines H1c2, H2c1, C1c1 and C2c1 are available at ArrayExpress under the accession number E-MTAB-13373 [116]. The POU5F1 and NANOG ChIP-seq data are available at GEO under the accession numbers GSE69646 [117] and GSE99627 [118], respectively. The PAX6 ChIP-seq data are available at the Genome Sequence Archive (GSA) of the China National Center for Bioinformation (CNCB-NGDC) under the accession number CRA002482 [119]. The Hi-C data were published under the DOI 10.1101/2025.03.11.642620 [61].
