# Supplementary material for: CroCoNet: a framework for the quantitative comparison of gene regulatory networks across species
Source: Genome Biol. 2026 Jul 15;27:228. doi: 10.1186/s13059-026-04152-5 (PMC13371563; doi:10.1186/s13059-026-04152-5)
Supplement: Supplementary file 1 — Additional file 1: Supplementary Figures S1-S14. [file 13059_2026_4152_MOESM1_ESM.pdf]

SUPPLEMENTARY FIGURES

CroCoNet: a framework for the quantitative comparison of gene regulatory networks across species

Anita Térmeg[1], Vladyslav Storozhuk[1], Zane Kliesmete[1], Fiona C. Edenhofer[1], Johanna Geuder[1], Tamina Dietl[2], Beate Vieth[1], Philipp Janssen[1], Daniel Richter[1], Boyan Bonev[2], Ines Hellmann[1]

[1]Anthropology and Human Genomics, Faculty of Biology, Ludwig-Maximilians-Universität München, 82152 Planegg, Germany

[2]Research Unit Brain Epigenomics, Helmholtz Center Munich, 81377 Munich, Germanyy

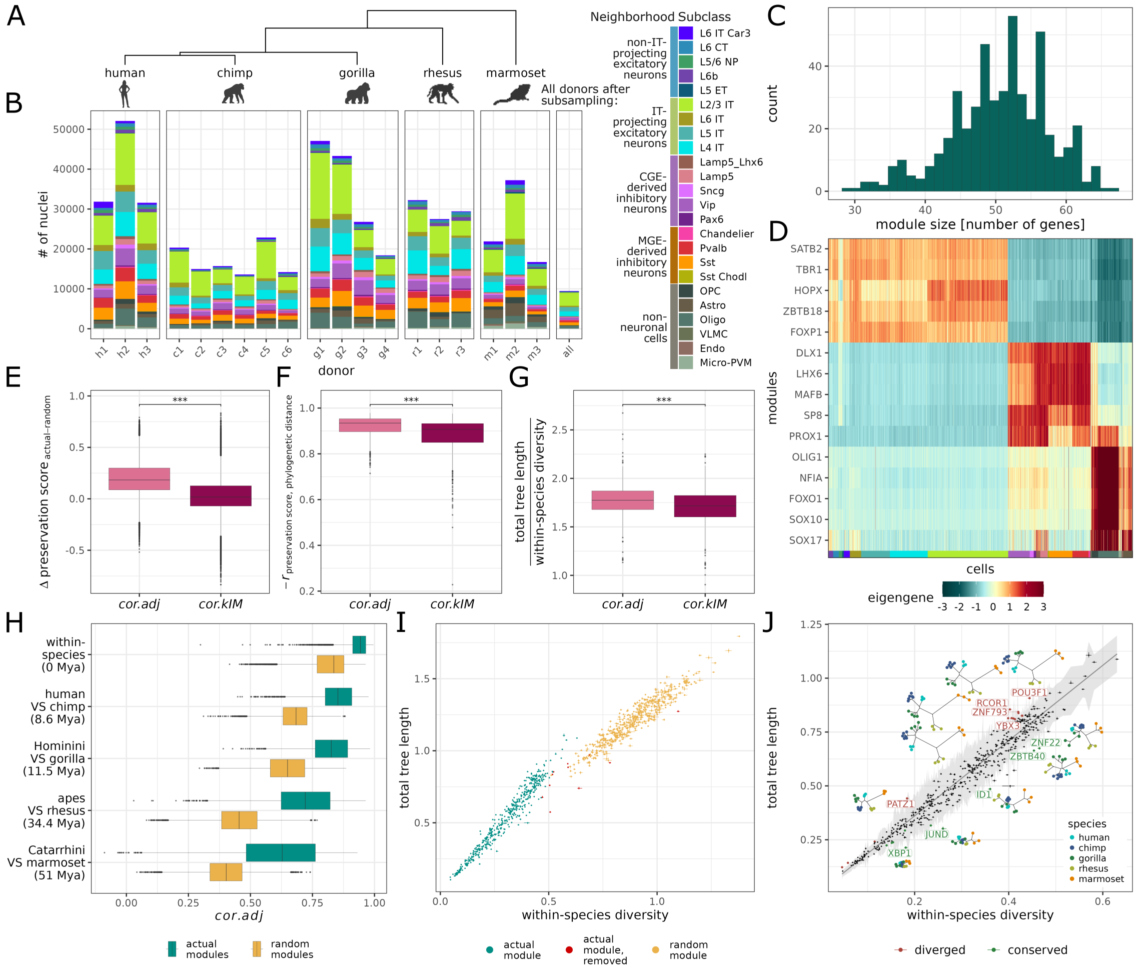

Fig. S1. CroCoNet analysis of a single-nucleus RNA-seq dataset profiling the middle temporal gyrus of five primate species. **A**) Phylogenetic tree of the five species. **B**) Number of nuclei and cell-type composition for each of the 19 donors after filtering. The last column shows the nuclei number and cell-type composition to which each donor was subsampled for network analysis. **C**) Distribution of module sizes after pruning. **D**) Module eigengenes for five excitatory neuronal, five inhibitory neuronal, and five non-neuronal markers. The eigengene calculation was based on the positively correlated targets. **E**) Difference in the *cor.kIM* / *cor.adj* scores between each actual and the corresponding random module. **F**) Inverted Pearson's correlation $(-r)$ between the *cor.kIM* / *cor.adj* scores and phylogenetic distance. **G**) Ratios of the total tree lengths and within-species diversities in the tree representations of the pruned modules, reconstructed based on the *cor.kIM* and *cor.adj* scores. **H**) Distribution of the *cor.adj* scores for the pruned and corresponding random modules, split by divergence time between the replicates compared. **I**) Total tree lengths and within-species diversities of the pruned and corresponding random modules, based on the trees reconstructed using the *cor.adj* scores. Eleven actual modules (in red) were not distinct enough from the random modules and were therefore excluded from the module divergence analysis. **J**) Quantification of overall module divergence. A weighted linear model was fitted to the total tree lengths and within-species diversities across all modules, and the 95% prediction interval of the regression line (shaded in gray) was calculated. Modules that fell above the upper bound or below the lower bound of the prediction interval were considered diverged and conserved, respectively. The five most conserved and five most diverged modules are labeled and displayed using the tree representations.

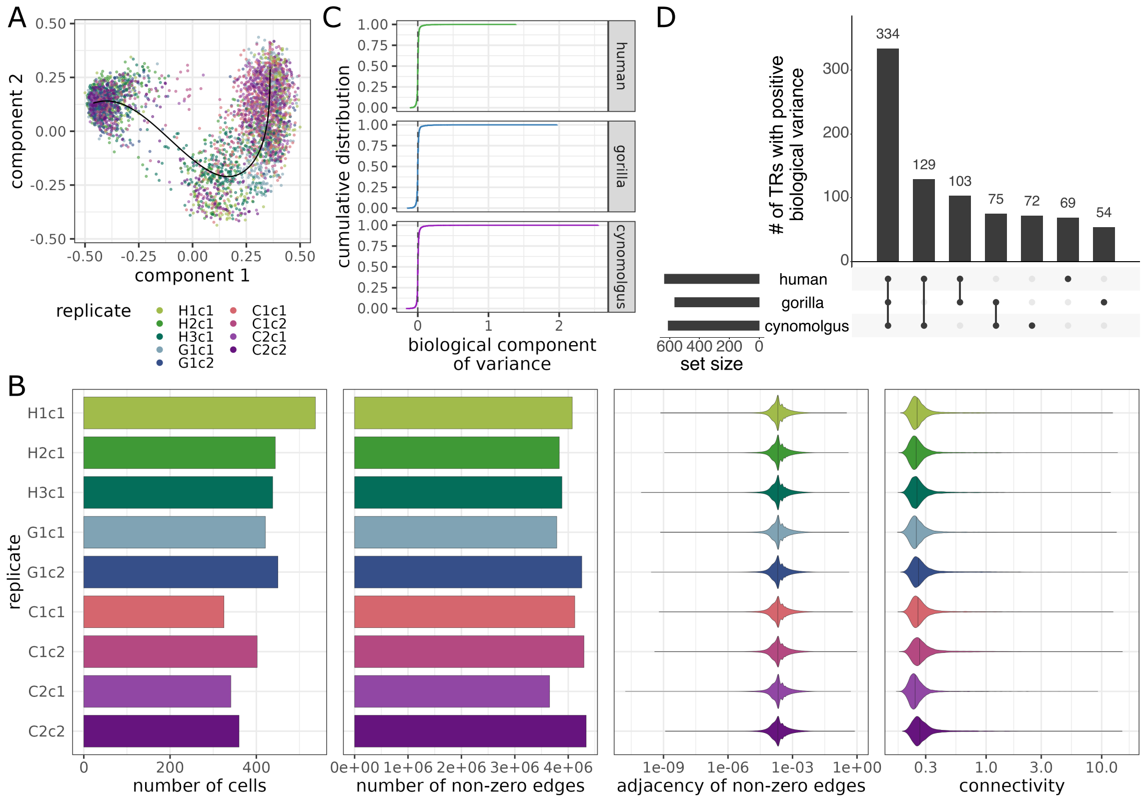

Fig. S2. Main characteristics of the replicate-wise networks and the construction of the consensus network. A) Pseudotime trajectory jointly inferred for all species, with cells colored by replicate. **B**) The number of cells that the network inference was based on, the number of non-zero edges, the distribution of adjacencies across all non-zero edges, and the distribution of connectivities across all genes for each replicate-wise network. **C**) Selection of relevant transcriptional regulators based on biological variance. The variance of the normalized gene counts was decomposed into technical and biological components, and TRs that had a positive biological variance in any of the species (cutoff indicated by the dashed line) were used as central regulators for the module assignment ($n = 836$). **D**) TRs with a positive biological variance shared among different subsets of the species.

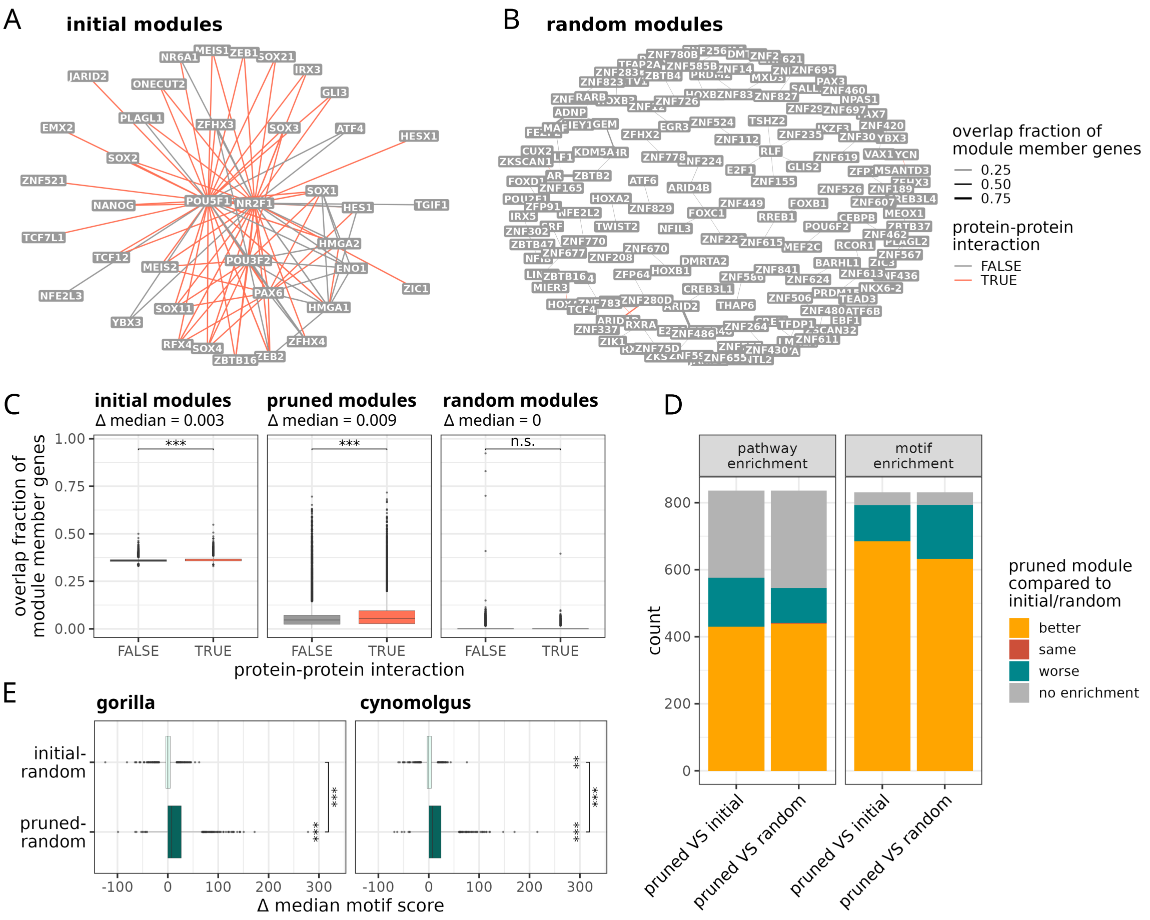

Fig. S3. Validation of the modules based on motif enrichment and target gene overlap. A-B) Overlaps between the initial and random modules and protein–protein interactions among their regulators. Nodes represent the initial/random modules associated with the regulators indicated in the labels, and edge weights represent the overlap fraction of module members. Only the 100 highest overlaps are shown. Red edges indicate that the regulators interact according to STRINGdb [28]. **C**) Overlap of module member genes for interacting versus non-interacting regulators. Interactions were defined based on STRINGdb [28]. The overlap between two pruned or initial modules was significantly higher if the corresponding regulators were interaction partners (Wilcoxon test, $n_1 = 17,670$, $n_2 = 331,360$, $p < 1 \cdot 10^{-16}$ in both cases), while the same was not observed for random modules ($p = 0.45$). The difference in median overlap (shown in the subtitles) was highest for the pruned modules. **D**) The number of modules that 1) improved, 2) worsened, 3) remained at the same level of enrichment, or 4) remained without any detected enrichment compared with the corresponding initial and random modules in terms of pathway and motif enrichment. **E**) Each target gene was scored for binding motifs of the module's central regulator in the gorilla and cynomolgus genomes (see Methods). The plot shows how the module-level summaries of these motif scores differ in the initial and pruned modules relative to the random modules for each species. Motif enrichment was calculated based on the human genome. **C**), **E**) Asterisks indicate significance: * $p < 0.05$, ** $p < 0.01$, *** $p < 0.001$.

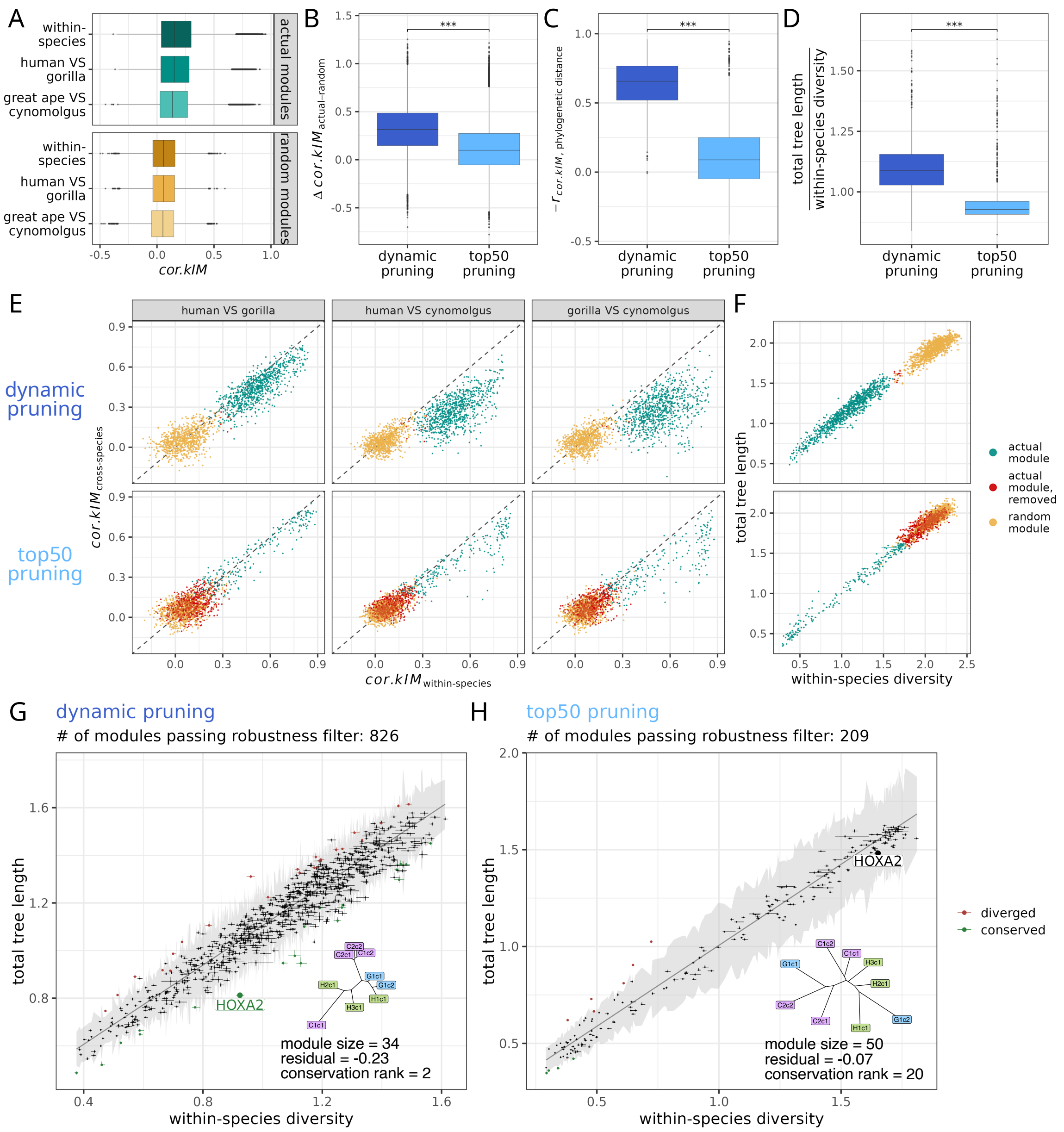

Fig. S4. Comparison of the dynamic and top50 pruning approaches. **A**) Distribution of *cor.kIM* for modules pruned using the top50 approach and the corresponding random modules, split by divergence time between the replicates compared. **B**) Difference in the *cor.kIM* scores between each actual and the corresponding random module, for the dynamic and top50 pruning. **C**) Inverted Pearson's correlation ($-r$) between *cor.kIM* and phylogenetic distance for the dynamic and top50 pruning. **D**) Ratios of the total tree lengths and within-species diversities in the tree representations of the pruned modules created by dynamic and top50 pruning. **E**) Within-species and cross-species *cor.kIM* scores of the pruned and corresponding random modules, split by pruning approach (top50 or dynamic) and species pair. Actual modules in red were not distinct enough from the random modules in terms of their tree characteristics and were therefore removed for the module divergence analysis. The separation between the actual and random modules is better, and the phylogenetic signal is stronger in case of the dynamic pruning (see B and C for quantification). **F**) Total tree length and within-species diversity of the pruned and corresponding random modules for the dynamic and top50 pruning approaches. The separation between the actual and random modules is better and the contribution of the within-species diversity (confounding factors) to the total tree length is lower in case of the dynamic pruning (see D for quantification). **G-H**) Network conservation of *HOXA2* based on dynamic and top50 pruning. In both cases, a linear model was fitted between the total tree lengths and within-species diversities across all modules, and the 95% prediction interval of the regression line (shaded in gray) was calculated. Modules that fell above the upper bound or below the lower bound of the prediction interval were considered diverged and conserved, respectively. Conservation of the *HOXA2* module was captured with the dynamic, but not with the top50 approach. **B-D**) Asterisks indicate significance based on Wilcoxon tests: * $p < 0.05$, ** $p < 0.01$, *** $p < 0.001$.

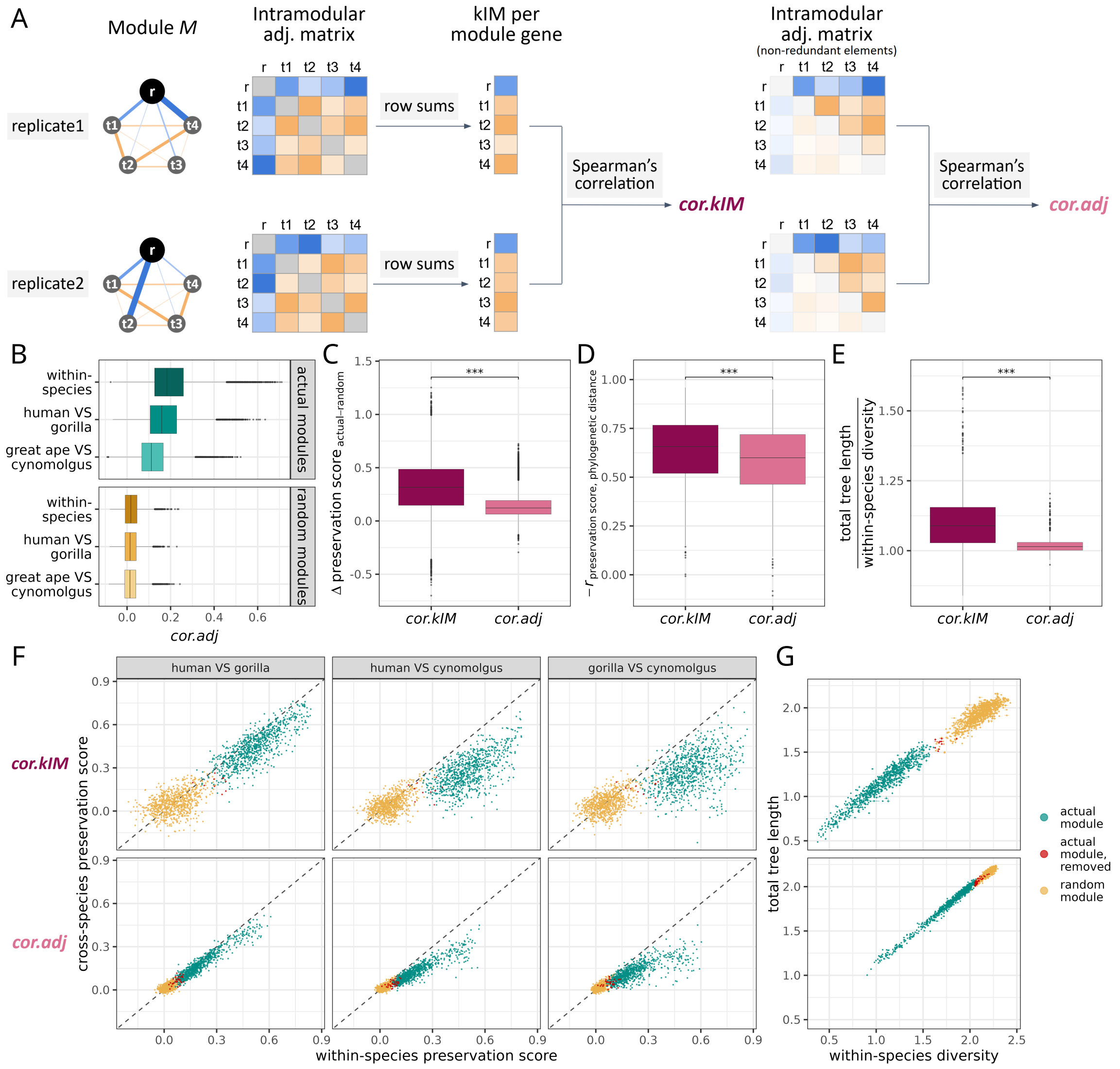

Fig. S5. Comparison of the preservation statistics *cor.kIM* and *cor.adj*. **A**) Calculation of *cor.kIM* and *cor.adj*. **B**) Distribution of *cor.adj* for the pruned and corresponding random modules, split by divergence time between the replicates compared. **C**) Difference in the *cor.kIM*/*cor.adj* scores between each actual and the corresponding random module. **D**) Inverted Pearson's correlation ($-r$) between *cor.kIM*/*cor.adj* and phylogenetic distance. **E**) Ratios of the total tree lengths and within-species diversities in the tree representations of the pruned modules, reconstructed based on the *cor.kIM* and *cor.adj* scores. **F**) Within-species and cross-species *cor.kIM* and *cor.adj* scores of the pruned and corresponding random modules, split by species pair. Actual modules in red were not distinct enough from the random modules in terms of their tree characteristics and were therefore removed for the module divergence analysis. The separation between the actual and random modules is better, and the phylogenetic signal is stronger for *cor.kIM* (see C and D for quantification). **G**) Total tree length and within-species diversity of the pruned and corresponding random modules, based on the trees reconstructed using the *cor.kIM* and *cor.adj* scores. The separation between the actual and random modules is better, and the contribution of the within-species diversity (confounding factors) to the total tree length is lower for *cor.kIM* (see E for quantification). **C-E**) Asterisks indicate significance based on Wilcoxon tests: * $p < 0.05$, ** $p < 0.01$, *** $p < 0.001$.

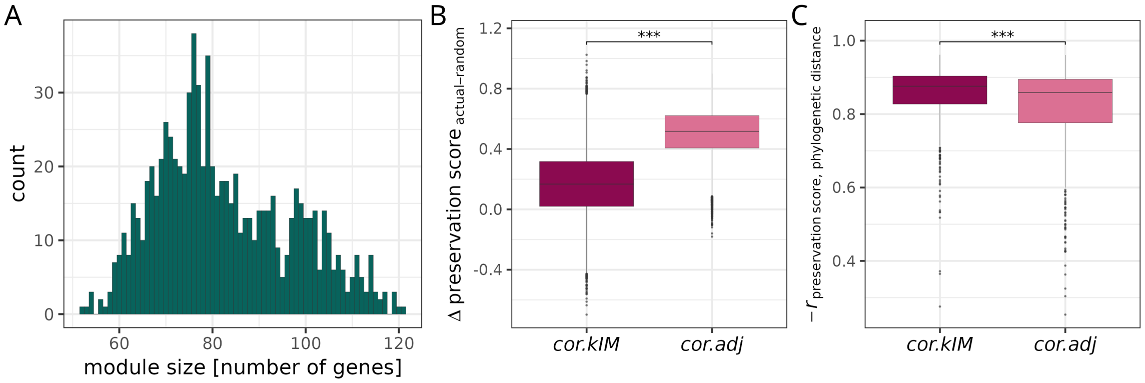

Fig. S6. Comparison of the preservation statistics *cor.kIM* and *cor.adj* in case of network inference with Spearman's correlation. A) Distribution of module sizes after pruning based on the Spearman networks. **B**) Difference in the *cor.kIM*/*cor.adj* scores between each actual and the corresponding random module. **C**) Inverted Pearson's correlation ($-r$) between *cor.kIM*/*cor.adj* and phylogenetic distance. Asterisks indicate significance based on Wilcoxon tests: * $p < 0.05$, ** $p < 0.01$, *** $p < 0.001$.

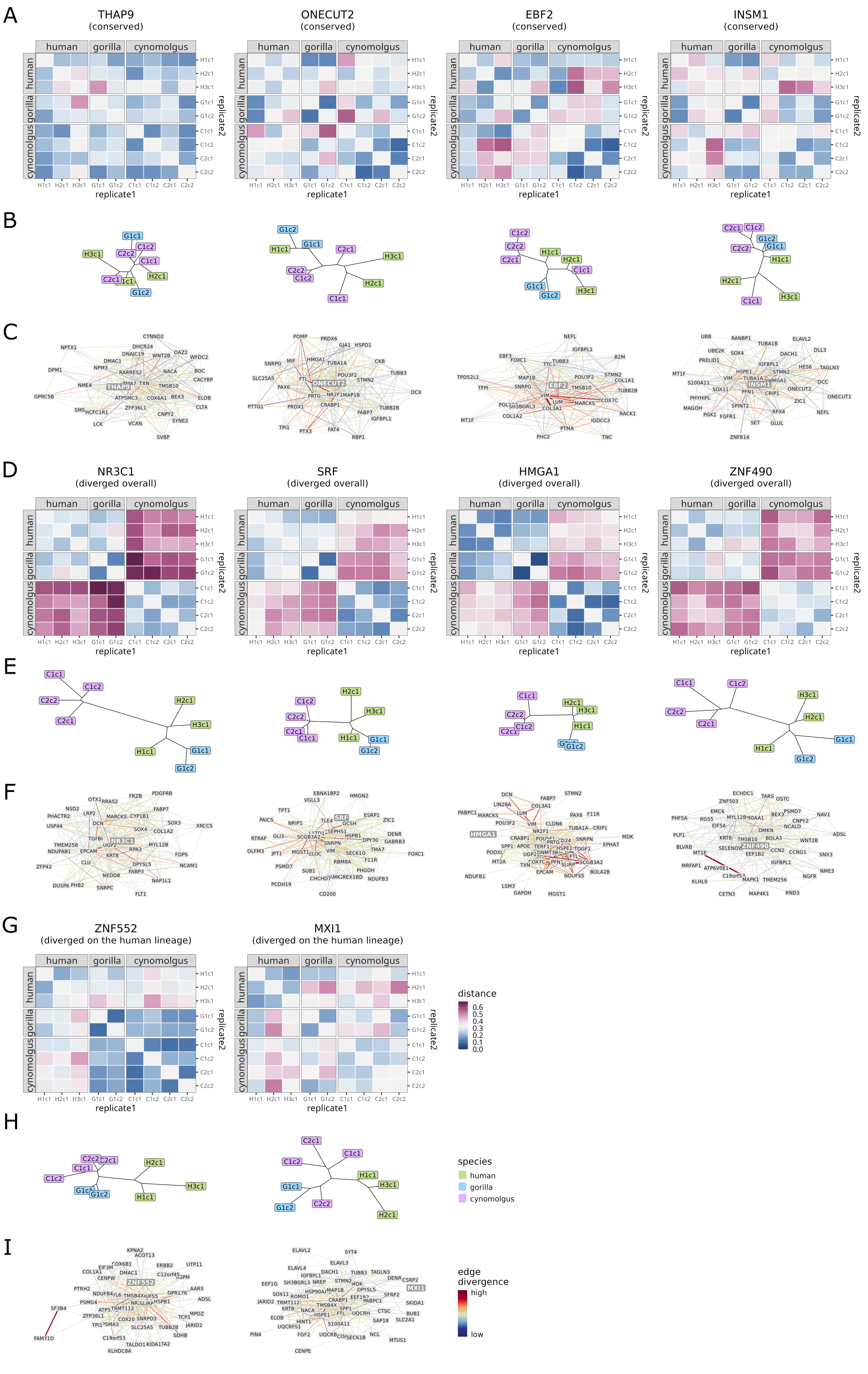

Fig. S7. Distance matrices, tree reconstructions, and network representations of the most conserved and most diverged modules. A) **D**) **G**) Distance matrices based on *cor.kIM* for the five most conserved modules, the five most diverged modules overall, and the three diverged modules on the human lineage, respectively (the three example modules in Fig. 3F-H are excluded). **B**) **E**) **H**) Neighbor-joining trees for the same modules as in A, D, and G, respectively. **C**) **F**) **I**) The 200 strongest connections of the same modules as in A, D, and G, respectively. The edge thickness represents the consensus edge weights (scaled per module) and edge color represents how different the mean edge weights are across the three species ($-\log_{10} F$ based on an ANOVA per edge).

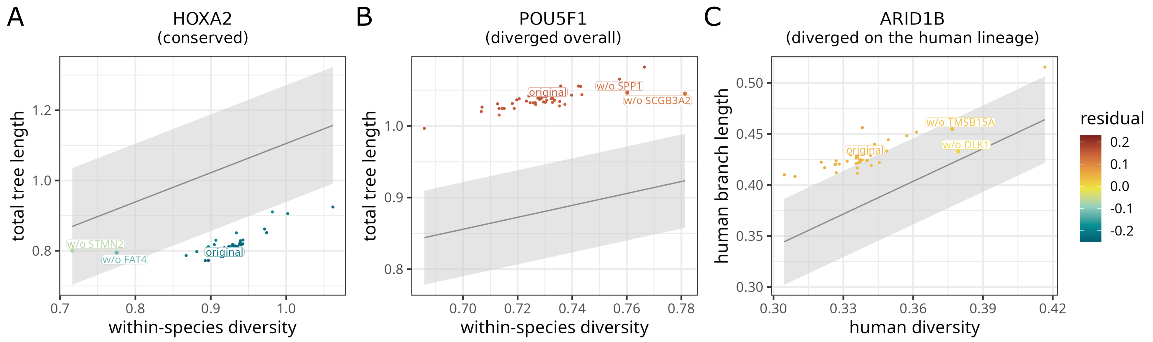

Fig. S8. Identifying the top-contributing genes within the *HOXA2*, *POU5F1*, and *ARID1B* modules using jackknifing. A-C) For *HOXA2* and *POU5F1*, the black line represents the regression line between the total tree lengths and within-species diversities of all modules, whereas for *ARID1B*, the black line represents the regression line between the human subtree lengths and human diversities of all human-monophyletic modules. The gray area marks the 95% prediction interval of the regression line in all cases. Each data point represents the original or a jackknifed version of the module, colored by its deviation from the regression line. The two jackknife versions with the highest reduction in the signal of conservation or divergence are labeled for each module. The labels indicate the target genes that have been removed. These are the top-contributing targets, i.e. in case of the conserved *HOXA2* module, they contribute the most to the signal of conservation, while in case of the diverged *POU5F1* and *ARID1B* modules, they contribute the most to the signal of divergence.

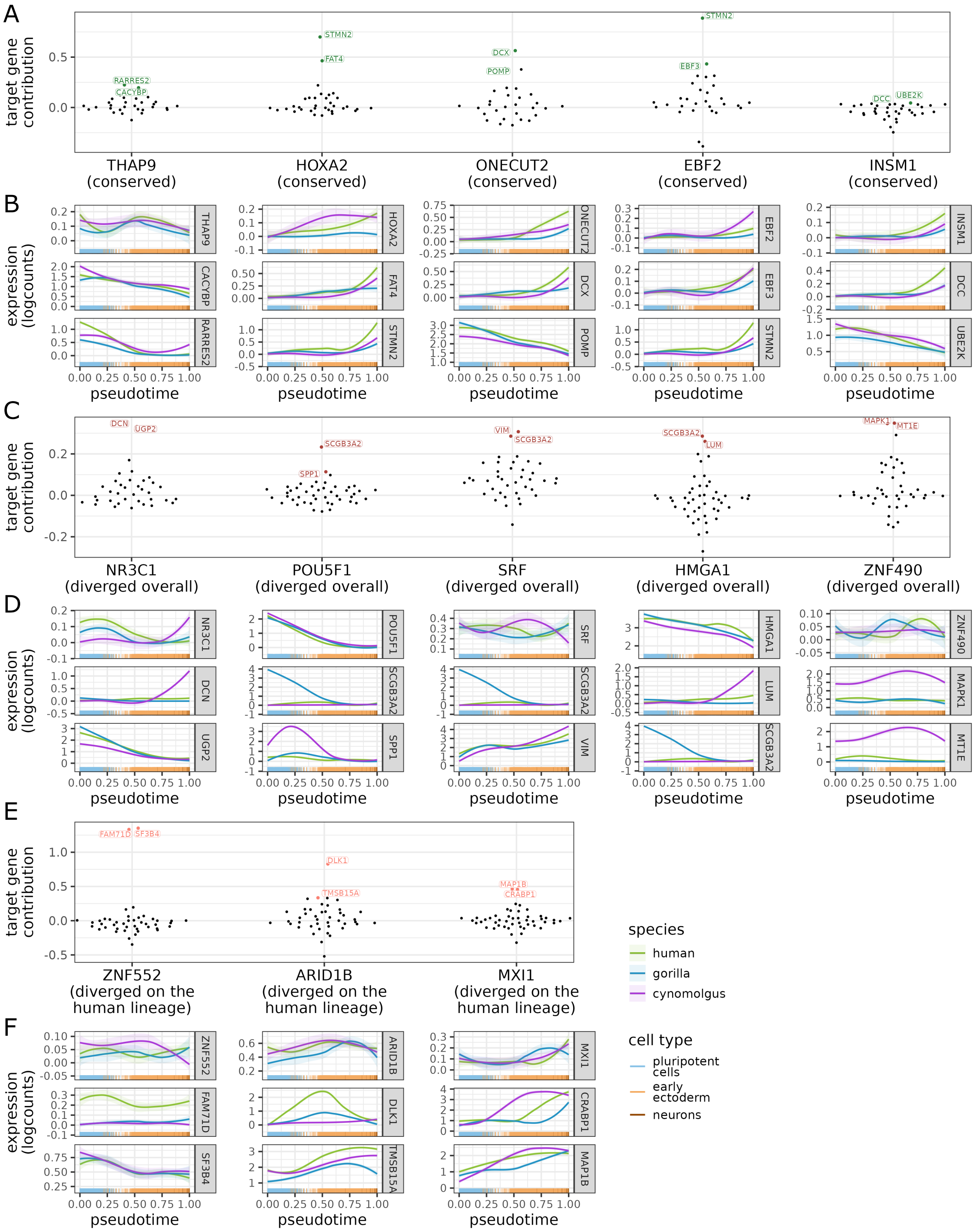

Fig. S9. Target gene contributions and expression profiles for the most conserved and most diverged modules. A) **C**) **E**) Target gene contributions for the five most diverged modules overall, the five most conserved modules, and the three diverged modules on the human lineage, respectively. For conserved modules, top-contributing targets drive the conservation signal, whereas for diverged modules, they drive the divergence signal. **B**) **D**) **F**) Expression profiles of the central regulator and its two targets with the highest contribution scores for the five most diverged modules overall, the five most conserved modules, and the three diverged modules on the human lineage, respectively.

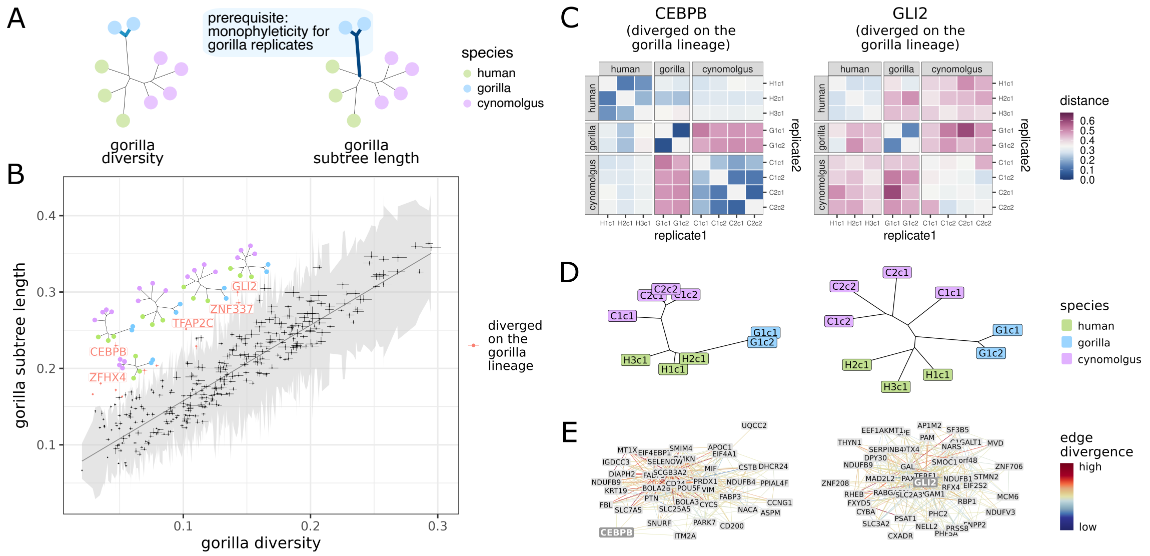

Fig. S10. Divergence on the gorilla and cynomolgus lineages. A) Gorilla diversity and gorilla subtree length. The gorilla subtree length is only defined if the tree is robustly monophyletic for the gorilla replicates. **B**) Quantification of module divergence on the gorilla lineage. A linear model was fitted between the gorilla subtree lengths and gorilla diversities across all gorilla-monophyletic modules, and the 95% prediction interval of the regression line (shaded in gray) was calculated. Modules that fell above the upper bound of the prediction interval were considered diverged. The five most diverged modules are labeled and depicted using the tree representations. **C**) Distance matrices based on *cor.kIM* for the two most diverged modules on the gorilla lineage. **D**) Neighbor-joining trees for the two most diverged modules on the gorilla lineage. **E**) The 200 strongest connections of the two most diverged modules on the gorilla lineage. The edge thickness represents the consensus edge weights (scaled per module) and edge color represents how different the mean edge weights are across the three species ($-\log_{10}F$ based on an ANOVA per edge).

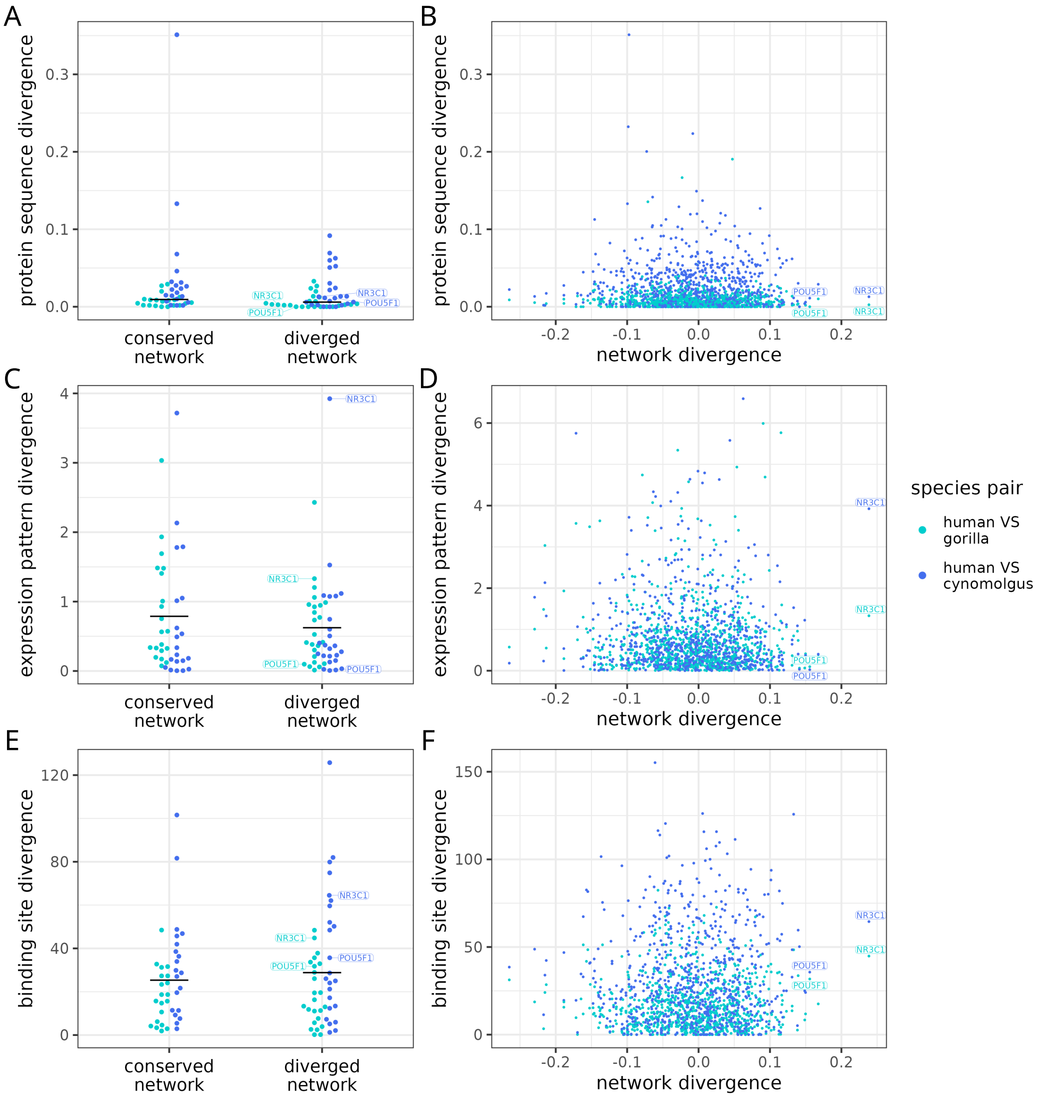

Fig. S11. Relationship between network divergence and the sequence, expression pattern and binding site divergence of the master regulator. A) Protein sequence divergence of the regulators associated with conserved and diverged modules, and **B**) protein sequence divergence of all regulators, plotted against their module's residual, a continuous measure of network divergence. Sequence divergence was quantified as the fraction of amino acid substitutions in the human-gorilla and human-cynomolgus protein sequence alignments. No significant difference was detected in this metric when comparing regulators with conserved and diverged networks (*sequence divergence* $\sim$ *network conservation category* + *phylogenetic distance*, $\beta = 0.01$, $p = 0.24$), nor when using continuous network divergence as a predictor (*sequence divergence* $\sim$ *residual* + *phylogenetic distance*, $\beta = 0.02$, $p = 0.09$). **C**) Expression pattern divergence of the regulators associated with conserved and diverged modules, and **D**) expression pattern divergence of all regulators, plotted against their module's residual. Expression pattern divergence was defined based on a DE analysis: $\log_2$ fold changes between the late and early pseudotime stages were estimated per species, then the absolute differences between the $\log_2$ fold-change values, normalized by mean expression, were calculated for each species pair. No significant difference was detected in this metric when comparing regulators with conserved and diverged networks (*expression pattern divergence* $\sim$ *network conservation category* + *phylogenetic distance*, $\beta = -0.16$, $p = 0.33$), nor when using continuous network divergence as a predictor (*expression pattern divergence* $\sim$ *residual* + *phylogenetic distance*, $\beta = -0.49$, $p = 0.12$). **E**) Binding site divergence of the regulators associated with conserved and diverged modules, and **F**) binding site divergence of all regulators, plotted against their module's residual. The binding site divergence was calculated based on scoring all annotated motifs of the regulators in the ATAC-seq peaks associated with their module member genes. For each regulator, the motif scores were summed per gene, then the absolute differences in these sum scores were calculated per species pair. Finally, these differences were summarized per species pair and module. No significant difference was detected in this metric when comparing regulators with conserved and diverged networks (*binding site divergence* $\sim$ *network conservation category* + *phylogenetic distance*, $\beta = 3.48$, $p = 0.47$), nor when using continuous network divergence as a predictor (*binding site divergence* $\sim$ *residual* + *phylogenetic distance*, $\beta = 2.11$, $p = 0.79$). However, the difference was significant between the five most conserved and five most diverged modules (Fig. 3P). **A-F**) *POU5F1* and *NR3C1*, the two main diverged examples, are labeled.

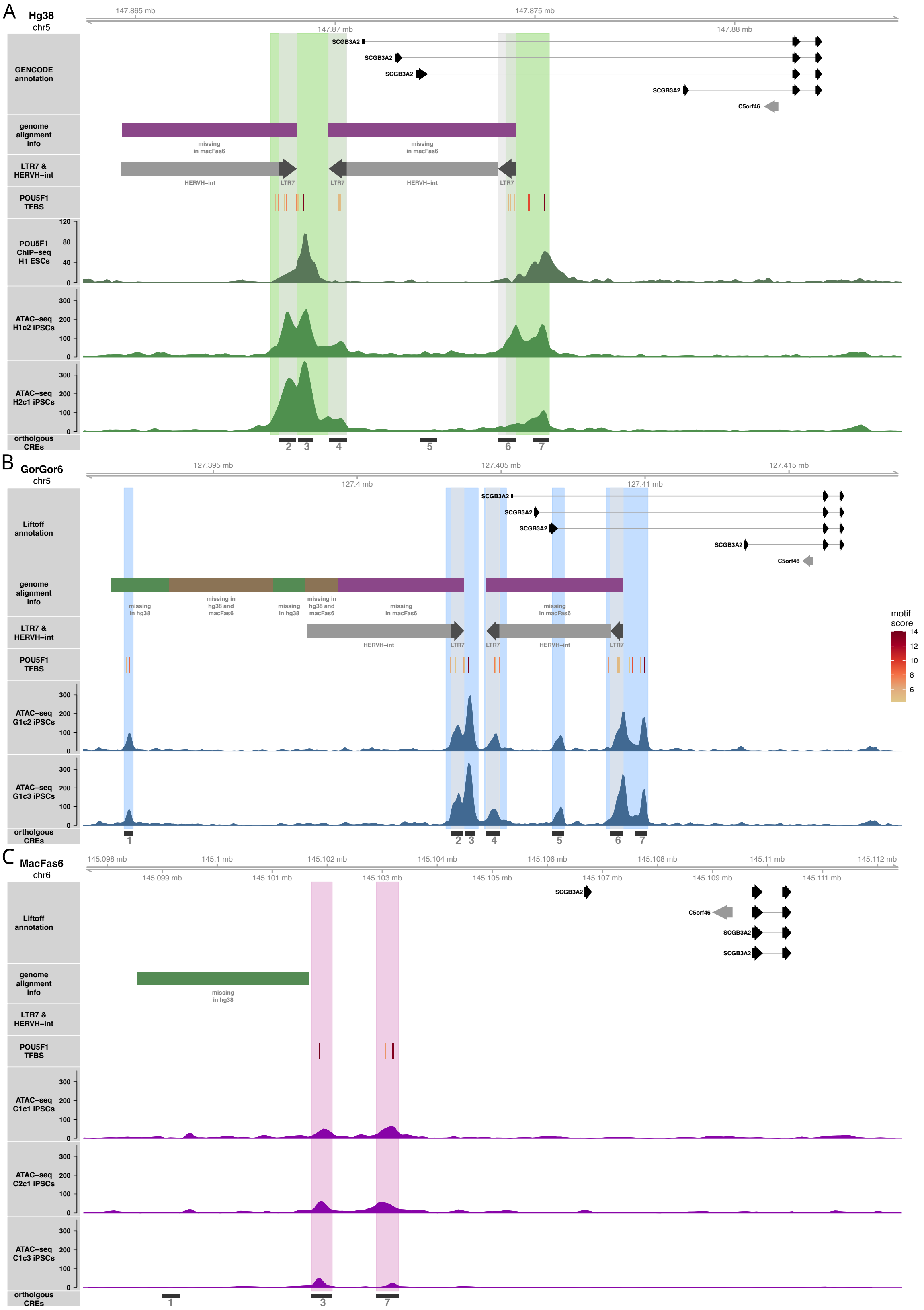

Fig. S12. The *SCGB3A2* locus in hg38, gorGor6, and macFas6. A) *SCGB3A2* and the region extending 7 kb upstream and 2 kb downstream in hg38. **B**) The region orthologous to A) in gorGor6. **C**) The region orthologous to A) in macFas6. The *ATAC-seq* tracks show data from different iPSC lines [81], and the *POU5F1 ChIP-seq* track shows data from H1-hESCs [41]. Gray shaded regions mark LTR7 elements, while colored regions mark ATAC-seq peaks for the respective species. The *genome alignment info* tracks highlight deletions and inversions larger than 1 kb in the other two genomes. The *POU5F1 TFBS* tracks show POU5F1 binding sites identified by Cluster-Buster within ATAC-seq peaks and LTR7 elements, with colors reflecting the motif scores. The *orthologous CREs* tracks show the orthology relationships among loci that were identified as candidate regulatory regions in at least one species (i.e. located in open chromatin or annotated as an LTR7 element).

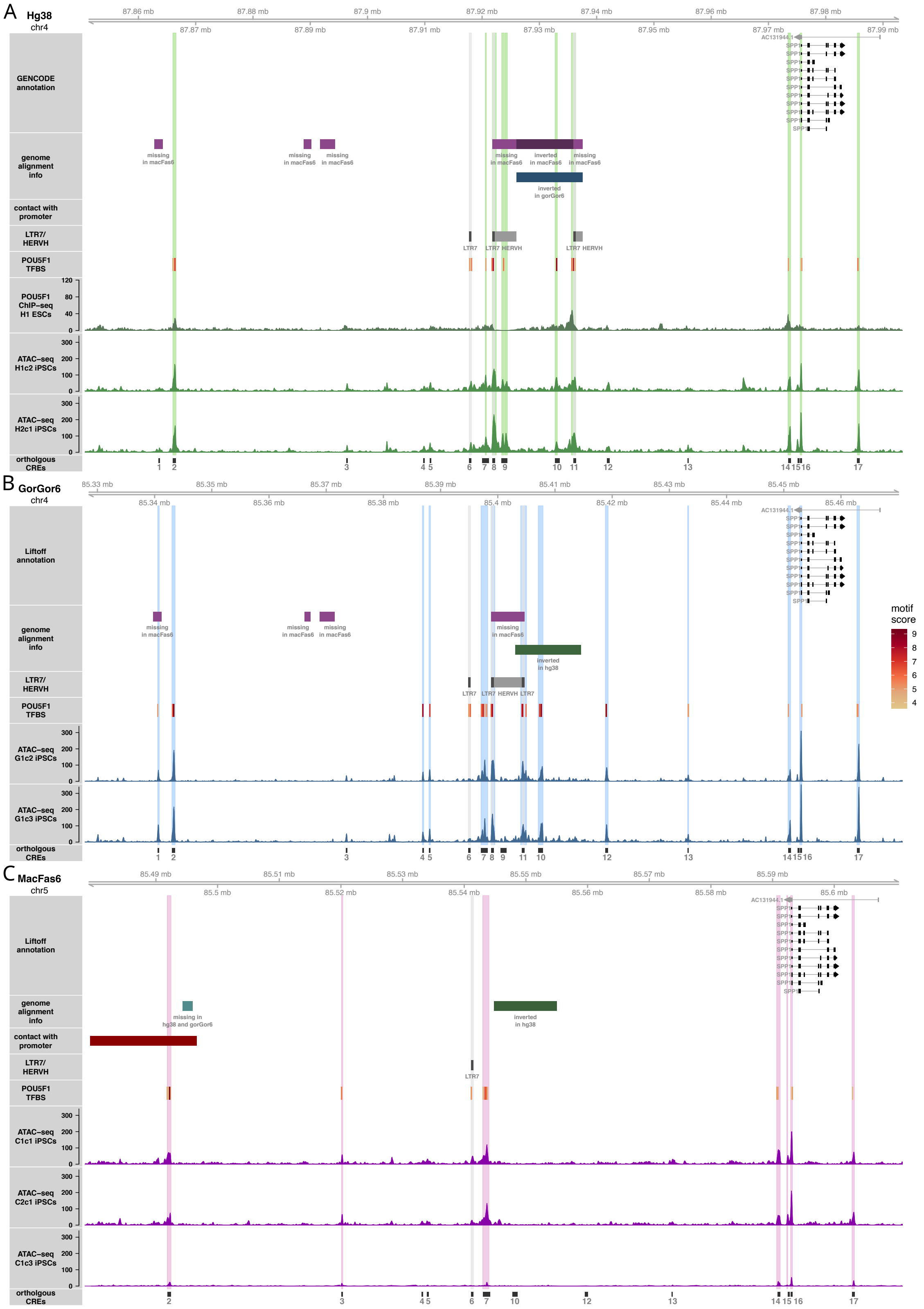

Fig. S13. The *SPP1* locus in hg38, gorGor6, and macFas6. A) *SPP1* and the region extending 125 kb upstream and 10 kb downstream in hg38. **B**) The region orthologous to A) in gorGor6. **C**) The region orthologous to A) in macFas6. The *ATAC-seq* tracks show data from different iPSC lines [81], and the *POU5F1 ChIP-seq* track shows data from H1-hESCs [41]. Gray shaded regions mark LTR7 elements, while colored regions mark ATAC-seq peaks for the respective species. The *genome alignment info* tracks highlight deletions and inversions larger than 1 kb in the other two genomes. The *POU5F1 TFBS* tracks show POU5F1 binding sites identified by Cluster-Buster within ATAC-seq peaks and LTR7 elements, with colors reflecting the motif scores. Regions in contact with the *SPP1* promoter (Hi-C score > 70) are indicated in the *contact with promoter* tracks. The *orthologous CREs* tracks show the orthology relationships among loci that were identified as candidate regulatory regions in at least one species (i.e. located in open chromatin or annotated as an LTR7 element).

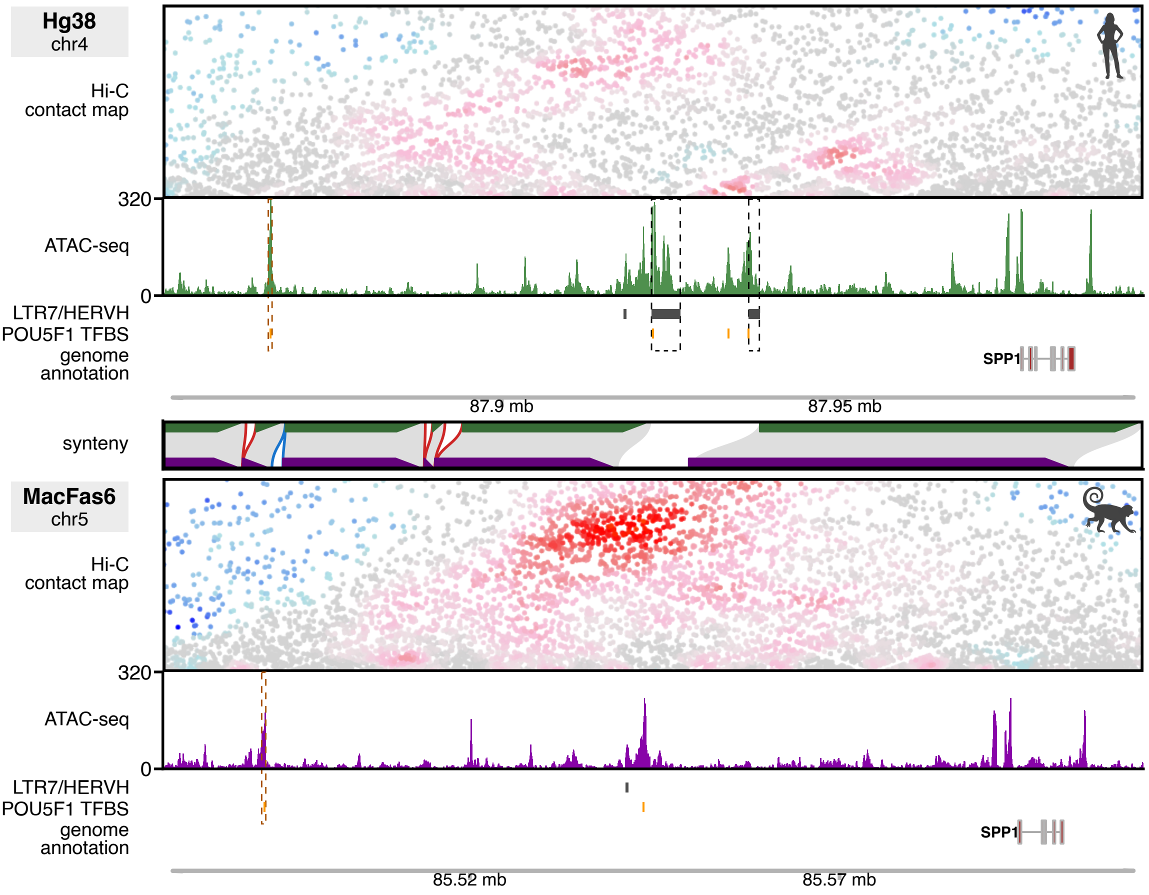

Fig. S14. Contact maps of the *SPP1* loci in hg38 and macFas6. Hi-C data from human and cynomolgus iPSCs [61] are shown for the region around *SPP1* in hg38 (−125 kb / +10 kb) and for the orthologous region in macFas6, respectively. The *ATAC-seq* tracks display the combined ATAC-seq coverage across all profiled iPSC lines of a species. The *POU5F1 TFBS* tracks highlight high-scoring POU5F1 binding sites identified by Cluster-Buster within ATAC-seq peaks and LTR7 elements. Only sites with a score in the top quantile across the human, gorilla, and cynomolgus *SPP1* loci are shown. Insertions (in blue) and deletions (in red) larger than 1 kb are marked in the *synteny* track based on the pairwise alignment of the two genomes. In the cynomolgus macaque genome, a strong chromatin contact can be observed between the *SPP1* promoter and a distal POU5F1-binding CRE (marked by the red dashed rectangle), while the orthologous CRE shows a much weaker interaction in the human genome. A possible explanation is that the LTR7/HERVH insertions in the human locus (marked by the black dashed rectangles) have disrupted the local 3D genome structure upstream of *SPP1*.
