# Supplementary material for: CroCoNet: a framework for the quantitative comparison of gene regulatory networks across species
Source: Genome Biol. 2026 Jul 15;27:228. doi: 10.1186/s13059-026-04152-5 (PMC13371563; doi:10.1186/s13059-026-04152-5)
Supplement: Supplementary file 3 — Additional file 3: Supplementary Methods. [file 13059_2026_4152_MOESM3_ESM.pdf]

SUPPLEMENTARY METHODS

CroCoNet: a framework for the quantitative comparison of gene regulatory networks across species

Anita Térmeg[1], Vladyslav Storozhuk[1], Zane Kliesmete[1], Fiona C. Edenhofer[1], Johanna Geuder[1], Tamina Dietl[2], Beate Vieth[1], Philipp Janssen[1], Daniel Richter[1], Boyan Bonev[2], Ines Hellmann[1]

[1]Anthropology and Human Genomics, Faculty of Biology, Ludwig-Maximilians-Universität München, 82152 Planegg, Germany

[2]Research Unit Brain Epigenomics, Helmholtz Center Munich, 81377 Munich, Germanyy

Neural differentiation experiment

Primate iPSCs (Additional File 2: Table S1) were cultured in StemFit + bFGF [79] and differentiated into NPCs via 9 days of dual-SMAD inhibition [85, 86]. On days 0, 1, 3, 5, 7, and 9, the progression of differentiation was validated by OCT4 and PAX6 staining, and cells were sampled for scRNA-seq. Libraries were prepared using the mcSCRB-seq protocol [87] and sequenced on an Illumina HiSeq 1500 instrument with 2×100 bp paired-end reads.

Processing of the differentiation dataset

After poly(A) trimming, the FASTQ data were processed using the zUMIs pipeline [88] that utilizes the aligner STAR [120]. We mapped all reads to three reference genomes: GRCh38/hg38 (GENCODE release 32), Kamilah_GGO_v0/gorGor6 (UCSC, Aug. 2019) and Macaca_fascicularis_6.0/macFas6 (ENSEMBL release 109). To reduce computational time, we removed contigs smaller than 150 kb from the gorGor6 genome. The gorilla and macaque GTF files were created by Liftoff of the hg38 annotation to the corresponding primate genomes [26], followed by removal of transcripts with partial mapping (<50%), low sequence identity (<50%) or excessive length (>100 bp difference and >2 length ratio).

We assigned the cells to the three species based on their barcode information and set filters for cell quality based on 1) the number of UMIs (2,000 < # of UMIs < 30,000 for human, 1,000 < # of UMIs < 32,000 for gorilla, and 1,000 < # of UMIs < 34,000 for cynomolgus), 2) the number of genes (>700), 3) the percentage of mitochondrial reads (<6%), and 4) the percentage of spike-in UMIs (<20%). We kept genes present in all three annotation files and expressed in >1% of the cells in all replicates of at least one species. In addition, we removed all mitochondrial, non-protein coding, ribosomal, histone and pseudogenes. Histone, ribosomal and pseudogenes were defined using the gene_group and locus_group annotations from the HGNC database [121]. We summed up the counts for paralog groups with >95% mean sequence identity based on the *Hsapiens gene ensembl* dataset of BioMart [122] and labeled each group by the first gene name among the paralogs in alphabetical order.

Count matrices were normalized using `scran::computeSumFactors` [89] and `scuttle::logNormCounts` [123]. For certain lines of analysis, we additionally applied per-batch scaling normalization using `batchelor::multiBatchNorm` [92]. We assigned cell type labels via SingleR [90] using a human embryoid body dataset [91] as reference. Non-neuronal cell types (Neural_Crest, Mesoderm, Endothelial_Cells, Endoderm), as well as the day 7 cells from the cynomolgus replicates C1c1 and C1c2 were removed for further analysis. For pseudotime inference, we regressed out the batch effects due to replicate differences using `batchelor::fastMNN` [92], then inferred a pseudotime trajectory using SCORPIUS [93] on a low-dimensional embedding of the batch-corrected counts (dist = 'spearman' and ndim = 25).

Network inference for the differentiation dataset

For the network inference, genes expressed in only 1 or 2 cells of a replicate were set to zero in those few cells as well to avoid spurious connections in the network. Raw counts were then normalized per replicate via `transformGamPoi::transformGamPoi`, using the randomized quantile residual transformation.

Based on the normalized counts, networks were reconstructed per replicate with GRNBoost2. For the list of transcriptional regulators, we used all 11,630 genes in the filtered count matrix. We ran GRNBoost2 [17] using the multiprocessing implementation of Arboreto (arboreto_with_multiprocessing.py) included in pySCENIC [95], 10 times for each replicate with 10 different seeds to make the results less dependent on the stochastic nature of the algorithm. We loaded and processed the networks using igraph [124].

First, we summarized the results per replicate. For each replicate, a given edge could appear up to 20 times (10 runs $\times$ 2 directions) in the output. For edges that appeared once or not at all, we set the edge weight to 0. For edges that appeared at least twice, we defined the edge weight as the mean importance score across the 20 occurrences; when an edge was not present for one of these 20 predictions, this was factored into the mean as a 0. The edge weights were then rescaled between 0 and 1 for all replicates together. To avoid false positive edges due to mapping errors, we set the weight of an edge to 0 if it connected two genes that had overlapping annotations in any of the species' genomes.

The adjacencies resulting from this network inference and processing approach were undirected (a single value per gene pair; regulator and target are not distinguished) and unsigned (strong activating and repressing interactions are both characterized by high adjacencies).

To investigate the effect of the network inference method on the best-performing preservation statistic, we also reconstructed networks for the neural differentiation dataset using Spearman's correlations per replicate (as described for the brain dataset in the Methods section "Network inference"). These networks were used only for Additional File 1: Fig. S6, all other analyses are based on the GRNBoost2 networks.

Mode of regulation

For an unsigned network (all examples in this manuscript), CroCoNet provides the option to store or add information on the mode of regulation, i.e. whether a connection is predicted to be activating or repressing. If the network inference method provides a sign to begin with (e.g. Spearman's correlation), it is simply saved independently of the edge weight for later use and propagated into the consensus network by aggregating the replicate-wise signs. For network inference methods that do not distinguish activating and repressing interactions (e.g. GRNBoost2, where the importance scores are inherently unsigned), the mode of regulation can be obtained *post hoc* for each consensus edge from gene–gene expression correlations. These correlations are calculated based on the log-normalized counts across all species, with replicates as blocks (`scran::correlatePairs` [89]). If the correlation is positive, the edge is regarded as activating, and if the correlation is negative, the edge is regarded as repressing.

In the downstream analysis, the consensus mode of regulation is used to split modules into activated and repressed targets of the central regulator for lines of analysis where this makes biological sense (e.g. pathway enrichment, eigengene calculation). The replicate-wise mode of regulation (if available) can be incorporated in the calculation of $cor.adj$ (see section "Preservation statistics").

Selection of the central regulators

We modeled the mean-variance relationship of the log-normalized counts and decomposed the variance into technical and biological components per species using the function `scran::modelGeneVar` [89], then identified the genes that had a positive biological variance in at least one species. We intersected these genes with transcriptional regulators that have at least one known motif based on the JASPAR 2024 core [19], JASPAR 2024 unvalidated [19] and IMAGE databases [20].

Module-level summaries of the pruning metrics

The replicate-wise networks were aggregated into a consensus network, and based on the consensus adjacencies, modules were assigned around each central regulator as described in the Methods.

The pruning metrics can be summarized per module as the mean regulator-target adjacency and the mean size-corrected kIM ($\overline{kIM}^{(q)}_{\text{size corr.}}$, Additional File 2: Table S5):

$$\overline{kIM}^{(q)}_{\text{size corr.}} = \frac{\sum_{i \in q} kIM_i^{(q)}}{n^2}, \tag{9}$$

where n is the module size, i.e. the total number of genes in the module.

Module overlaps

In the neural differentiation dataset, the overlap between the member genes of two modules q_1 and q_2 was quantified using the overlap fraction:

$$\text{overlap}(q_1, q_2) = \frac{|q_1 \cap q_2|}{\min(|q_1|, |q_2|)} \tag{10}$$

Protein-protein interactions between the regulators were identified using STRINGdb's human database [28] with a minimum score threshold of 200. We found that interacting regulators had significantly higher target gene overlaps between their pruned modules than non-interacting regulators (Wilcoxon test, $n_1 = 17,670$, $n_2 = 331,360$, $p < 1 \cdot 10^{-16}$, Additional File 1: Fig. S3C). This was

also the case for the initial modules, although to a lesser extent, and was not observed at all for the random modules.

Pathway enrichment analysis

For the neural differentiation dataset, we performed pathway enrichment analysis on the activated targets of the initial, pruned, and random modules. To quantify enrichment, we used Reactome [45, 46], with all genes in the network as universe. We regarded pathways with an adjusted p-value <0.1 (Benjamini-Hochberg FDR correction) as significant. As a module-level summary metric, we calculated the fraction of module genes that are annotated to any of the enriched pathways.

Module eigengenes

We summarized the expression profile of each module using the concept of module eigengenes. We selected the module genes that showed a positive correlation to the central regulator, subsetted the normalized count matrix for these genes, then calculated the first principal component of this matrix aligned with the average expression as implemented by `WGCNA::moduleEigengenes` [44].

ATAC-seq data

Part of the ATAC-seq dataset used for motif enrichment analysis was previously published [81]; here we describe both the published and unpublished work. ATAC-seq data were collected from seven primate cell lines (Additional File 2: Table S1): two human iPSC clones of two individuals (H1c2, H2c1), two gorilla iPSC clones of one individual (G1c2, G1c3) and three cynomolgus iPSC clones of two individuals (C1c1, C1c3, C2c1). Two human clones (H1c2, H2c1) and two cynomolgus clones (C1c1, C2c1) were differentiated as described in section ”Neural differentiation experiment” and ATAC-seq data were also collected from NPCs after differentiation. All libraries were generated using the Omni-ATAC protocol [100] with minor modifications.

We mapped reads to the same reference genomes as for the scRNA-seq data using BWA-MEM2 [101]. We called peaks jointly for all replicates per cell type and species using genrich [102] in a

two-step process. In the first step, a blacklist was created for each genome by taking peaks with unusually high widths and signal values. In the second step, peak calling was repeated using these blacklists. To gain information about accessibility in gorilla NPCs, the orthologous regions for the human NPC peaks were identified in the gorGor6 genome using the UCSC LiftOver tool [103].

Long-read RNA-seq data

Total RNA was isolated from samples of seven primate cell lines (Additional File 2: Table S1): two human iPSC samples (H2c1, H2c2), a human NPC sample (H1c2), a gorilla iPSC sample (G1c1), a gorilla NPC sample (G1c2), a cynomolgus iPSC sample (C1c1), and three cynomolgus NPC samples (two different passages of C1c1, and C1c2). For all cell lines, 50 ng RNA were reverse-transcribed using the PCR-cDNA Barcoding Kit SQK-PCB109 (Oxford Nanopore Technologies). Purified barcoded cDNAs were pooled and sequenced on a PromethION R9.4.1 flow cell (Oxford Nanopore Technologies).

Reads were mapped to the same reference genomes as for the scRNA-seq data using minimap2 [125]. Aligned reads were converted into transcript models using the pinfish pipeline (Oxford Nanopore Technologies). Gene names were assigned to the transcripts if >60% of their base pairs across all exons overlapped with exons of the respective gene in the GENCODE or Liftoff annotation.

Peak-to-gene associations

We identified the transcriptional start sites (TSS) of active genes in human, gorilla, and cynomolgus iPSCs and NPCs. TSS were defined as the 5'-most positions of transcript models derived from the GENCODE or Liftoff annotations, and from long-read RNA-seq data of the species and cell type of interest. TSS within 100 bp of each other were subsequently merged. A gene was regarded as active if it fulfilled at least one of the following criteria: 1) the TSS was identified based on long-read RNA-seq data, 2) the TSS overlapped with an ATAC-seq peak of the corresponding species and cell type, 3) the gene was expressed in >5% of the cells and had a mean log-expression of >0.05 in the scRNA-seq data in the corresponding species and cell type.

For each cell type and species, ATAC-seq peaks were associated with genes based on their distance to TSSs as described in [81]. Peaks within 2 kb of a TSS were regarded as promoters and were associated with all TSSs within that distance. All other peaks within 20 kb of a TSS were considered enhancers and were associated with the two closest TSSs in each direction, unless the distance to one TSS was >10× smaller than to the other TSS; in that case, only the closest TSS was kept.

Next, peak-to-gene associations from iPSCs and NPCs of the same species were unified. If an iPSC and NPC peak overlapped by more than 50%, and the associated genes (in either direction) differed between cell types, the gene closest to the peak was assigned in both cases.

Motif scoring

For each of the 836 regulators in the neural differentiation dataset, we identified known binding motifs and their position weight matrices from the JASPAR 2022 vertebrate core [104], JASPAR 2022 vertebrate unvalidated [104] and IMAGE databases [20]. We scored all motifs of a regulator in all peaks associated with the genes in its initial, pruned and random modules using Cluster-Buster [105]. We used a padding of 500 bp and a range of 10,000 bp to calculate local nucleotide abundances and kept all hits without a motif score threshold.

Next, we collapsed redundant motif hits. If several hits on the same strand, within the same peak and for the same regulator overlapped, we kept only the one with the highest score. If an iPSC and NPC peak of the same species overlapped, we merged them and performed this summarization across the entire union peak. Finally, we summarized the binding potential per gene, regulator and species (G) by summing up the non-redundant motif scores (S) across all associated peaks in the given species:

$$G_{g,r,s} = \sum_{p \in Peaks(g)} S_{p,r,s}, \tag{11}$$

where g is the gene, r is the regulator, s is the species, and $Peaks(g)$ is the set of peaks associated with g. For genes that had associated peaks but no motif hits, this summarized score was regarded as 0, while genes that had no associated peaks to begin with were excluded from the downstream

analysis.

Module validation using motif scores

To compare the random, initial and pruned modules of the neural differentiation dataset in terms of binding potential of the regulator, we further summarized the scores per module and species (M) by taking the median across all module member genes (Additional File 2: Table S5):

$$M_{r,s,t} = \underset{g \in Module(r,t)}{\text{median}} G_{g,r,s}, \tag{12}$$

where t is the module type (initial, pruned or random), and $Module(r, t)$ is the set of genes in the module of type t assigned to regulator r.

Then we calculated the Δ motif scores between the corresponding initial and random as well as between the corresponding pruned and random modules. Finally, we performed paired two-sided Wilcoxon tests per species to compare 1) the initial and random scores ($M_{r,s,\text{initial}}$ vs. $M_{r,s,\text{random}}$), 2) the pruned and random scores ($M_{r,s,\text{pruned}}$ vs. $M_{r,s,\text{random}}$), and 3) the pruned-random and initial-random Δ scores ($M_{r,s,\text{pruned}} - M_{r,s,\text{random}}$ vs. $M_{r,s,\text{initial}} - M_{r,s,\text{random}}$).

Cross-species differences in motif scores

To investigate cross-species changes in binding potential, we calculated the differences in gene-wise motif scores between pairs of species (ΔG, Additional File 2: Table S5):

$$\Delta G_{g,r,s1,s2} = |G_{g,r,s1} - G_{g,r,s2}| \tag{13}$$

Then we summarized the ΔG values per pruned module and species pair (ΔM) by taking the median across all genes to obtain a measure of binding site divergence between the two species:

$$\text{binding site divergence}_{s1,s2} = \underset{g \in Module(r,\text{pruned})}{\text{median}} \Delta G_{g,r,s1,s2} \tag{14}$$

Finally, to test whether regulators with conserved and diverged network modules differ in terms of their binding site divergence, we restricted the analysis to non-redundant species comparisons (human-gorilla and human-cynomolgus only) and fitted the following linear model across the regulators whose modules were classified as conserved or diverged in the CroCoNet analysis:

$$\text{binding site divergence} \sim \text{network conservation category} + \text{phylogenetic distance} \tag{15}$$

Based on the model, regulators of diverged modules tend to be associated with higher binding site divergence than regulators of conserved modules, but this difference was not significant ($\beta = 3.48$, $p = 0.47$). When comparing the five most diverged and five most conserved modules only, this difference is more pronounced, reaching statistical significance ($\beta = 14.9$, $p = 0.024$).

As a complementary analysis, we also fitted the following linear model across all regulators (for non-redundant species comparisons only):

$$\text{binding site divergence} \sim \text{network divergence (residual)} + \text{phylogenetic distance} \tag{16}$$

The continuous measure of network divergence (residual) showed a positive but non-significant association ($\beta = 2.11$, $p = 0.79$).

ChIP-seq enrichment

We validated the modules of three well-studied regulators, *POU5F1*, *NANOG*, and *PAX6*, based on published ChIP-seq data. In line with the expression profiles of these modules, we used a dataset profiling H1 human embryonic stem cells (hESCs) for POU5F1 [41], a dataset profiling H9 hESCs for NANOG [42], and a dataset profiling H9-derived NPCs for PAX6 [43]. We mapped reads from all three datasets to the hg38 reference genome using bowtie [126]. In the case of the POU5F1 dataset that included two ChIP replicates, we called peaks jointly for these replicates using genrich [102]. For NANOG and PAX6, we called peaks using MACS3 [127] with $q = 0.01$. In all cases, we excluded

problematic regions based on a blacklist provided by ENCODE [128].

To check for ChIP-seq enrichment near module member genes, we defined the cis-regulatory landscape of each gene as the set of associated ATAC-seq peaks, restricted to peaks from iPSC lines for POU5F1 and NANOG enrichment, and peaks from NPC lines for PAX6 enrichment. We found that POU5F1, NANOG, and PAX6 ChIP-seq peaks overlapped with the cis-regulatory landscapes of the respective module genes significantly more often than with the cis-regulatory landscapes of all other genes (two-sided Fisher's exact test, odds ratio = 5.72 and $p = 3 \cdot 10^{-6}$ for POU5F1, odds ratio = 4.11 and $p = 3 \cdot 10^{-5}$ for NANOG, and odds ratio = 5.58 and $p = 0.02$ for PAX6).

Preservation statistics

To introduce the two preservation statistics that CroCoNet relies on, let q denote a consensus module with n_q genes, and let $A^{[net](q)}$ denote its intramodular adjacency matrix, i.e. the $n_q \times n_q$ dimensional subset of the entire adjacency matrix in the network *net* that contains only the module genes. In addition, let *vectorizeMatrix()* denote a function that takes a matrix as input and returns its non-redundant elements (i.e. the upper triangular elements in case of a symmetric matrix) in a vector form.

Then the correlation of adjacencies (*cor.adj*) and correlation of intramodular connectivities (*cor.kIM*) [8] can be calculated between the networks of two replicates (*net1* and *net2*) as follows:

$$cor.adj^{(q)} = \mathrm{cor}(\mathrm{vectorizeMatrix}(A^{[net1](q)}), \mathrm{vectorizeMatrix}(A^{[net2](q)})), \tag{17}$$

$$cor.kIM^{(q)} = \mathrm{cor}(kIM^{[net1](q)}, kIM^{[net2](q)}), \tag{18}$$

If the analysis is done with unsigned networks but the network inference method originally provided signed edge weights, CroCoNet provides the option to reintroduce these signs specifically for the calculation of *cor.adj* (this was done for the Spearman networks inferred on the brain data). The rationale behind this is that an edge known to be activating in one species and repressing in another

should not contribute to a high preservation. This option is not offered for cor.kIM, because kIM reflects the relative importance of a module member, which is increased by both strong activating and strong repressing edges equally.

Edge divergence

For each edge in the network, we calculated an edge divergence score based on the edge weights inferred in the different species and replicates. We performed a one-way ANOVA with the species as groups and the replicates as observations, calculated the corresponding F-statistic and defined the edge divergence as follows:

$$\text{edge divergence} = -\log_{10}F, \tag{19}$$

Processing of the *POU5F1* CRISPRi data

Reads were processed using 10x CellRanger (version 7.0.0, https://support.10xgenomics.com/single-cell-gene-expression/software/pipelines/latest/what-is-cell-ranger). We mapped reads of the gene expression (GEX) library to the hg38 and macFas6 reference genomes extended by the sequence of the dCas9-construct, and reads of the CRISPR library to a custom reference created using the gRNA protospacer sequences. The reads were demultiplexed into species and individuals in a two-step process using cellsnp-lite [129] and vireo [130], based on a list of single nucleotide polymorphisms (SNPs) compiled from bulk RNA-seq data of the wild-type cell lines.

We kept cells that passed basic quality control, carried a single dominant gRNA (i.e. all other detected gRNAs in the cell made up <10% of gRNA UMIs and <1000 UMIs combined), and had >10 UMIs supporting the dominant gRNA. We excluded gRNAs from a species if they were detected in <20 cells in either individual of that species. To remove control gRNAs with an unwanted transcriptomic effect, we performed differential expression (DE) analysis with limma-trend [131], comparing each control gRNA against all others. We iteratively removed any gRNA that had more DE genes (adjusted p-value <0.05) than $mean + 3\sigma$ across the DE gene counts of all control gRNAs. We repeated this

process until all remaining gRNAs fell within this range. After these filtering steps, we retained 1,635 *POU5F1*-perturbed cells (5 gRNAs) and 16,981 control cells (39 gRNAs) for the human, and 1,482 *POU5F1*-perturbed cells (6 gRNAs) and 16,421 control cells (37 gRNAs) for the cynomolgus (Additional File 2: Tables S8-S9).

We kept genes that could be transferred from the hg38 to the macFas6 genome via Liftoff [26] and were expressed in at least one condition (perturbed or control) of at least one species (>10% of cells and >10 cells) robustly across replicates. In addition, we excluded non-protein coding, mitochondrial, and Y-chromosomal genes. This filtering approach retained 10,413 genes.

To characterize the pluripotency state of the cells, we calculated a transcriptome-based stemness index for each cell. We applied a one-class logistic regression model trained on pluripotent stem cell samples [132]. The resulting scores were scaled between 0 and 1 across both species.

For visualization, we performed normalization, feature selection and PCA dimensionality reduction using Seurat v5 [133], then integrated the data across individuals and batches per species using Harmony [134] with $\theta = 3$, and finally applied UMAP dimensionality reduction using Seurat v5.

To quantify *POU5F1* downregulation, we calculated a % knockdown (% KD) value for each gRNA:

$$\% \text{ KD} = \left(1 - \frac{\text{mean expression (perturbed)}}{\text{mean expression (control)}}\right) \cdot 100 \tag{20}$$

Protein sequence divergence

The hg38-gorGor6 and hg38-macFas6 protein alignments created by TOGA [51] were downloaded from `http://genome.senckenberg.de/download/TOGA/human_hg38_reference/Primates/`. For each of the 836 regulators in the neural differentiation dataset, we identified the longest canonical coding sequence (CCDS), or if no CCDS were annotated, the longest coding sequence (CDS) in the hg38 GENCODE v32 annotation. Based on the protein alignments corresponding to these CDS, we calculated a measure

of protein sequence divergence as follows (Additional File 2: Table S5):

$$\text{protein sequence divergence} = \frac{n_{\text{mismatches}}}{n_{\text{aligned}}}, \tag{21}$$

where n_{aligned} is the number of aligned amino acid positions where both species have a non-gap character, and $n_{\text{mismatches}}$ is the number of mismatches among those aligned positions.

To test whether regulators with conserved and diverged network modules differ in terms of their protein sequence divergence, we fitted the following linear model across the regulators whose modules were classified as conserved or diverged in the CroCoNet analysis:

$$\text{protein sequence divergence} \sim \text{network conservation category} + \text{phylogenetic distance} \tag{22}$$

Based on the model, there is no significant difference in protein sequence divergence between regulators with conserved and diverged modules ($\beta = 0.01$, $p = 0.24$).

As a complementary analysis, we also fitted the following linear model across all regulators:

$$\text{protein sequence divergence} \sim \text{network divergence (residual)} + \text{phylogenetic distance} \tag{23}$$

The continuous measure of network divergence (residual) was also not a significant predictor ($\beta = 0.02$, $p = 0.09$).

Expression pattern divergence

Based on pseudotime (pt), cells of the neural differentiation dataset were binned into three stages: early/iPSC ($pt \leq 0.25$), intermediate ($0.25 < pt \leq 0.75$), and late/NPC ($pt > 0.75$). The data were subsampled to an equal number of cells across the three species in each of the stages (early stage: 346 cells/species, intermediate stage: 155 cells/species, late stage: 223 cells/species). During the subsampling, we ensured that both the number of cells and the pseudotime distribution of each stage

were as similar as possible across species.

Using dream [107], we fitted the following mixed-effects model to the log-normalized counts of each gene:

$$\text{gene expression} \sim 0 + \text{species\_stage} + (1 \mid \text{replicate}), \tag{24}$$

where "species_stage" denotes the combination of species and stage, which can take up nine different values (3 species $\times$ 3 stages).

Based on the model, we estimated $\log_2$ fold changes between the late and early stages in each species. Subsequently, we calculated the absolute differences between the $\log_2$ fold change values per species pair (corresponding to estimates of a species:stage interaction term) normalized by the mean expression to obtain a measure of expression pattern divergence (Additional File 2: Table S5):

$$\text{expression pattern divergence}_{s1,s2} = \frac{|\log_2 \text{FC}_{\text{late-early, } s1} - \log_2 \text{FC}_{\text{late-early, } s2}|}{\text{mean expr.}_{s1,s2}} \tag{25}$$

To test whether regulators with conserved and diverged network modules differ in terms of their expression pattern divergence, we subsetted non-redundant species comparisons (human-gorilla and human-cynomolgus only) and fitted the following linear model across the regulators whose modules were classified as conserved or diverged in the CroCoNet analysis:

$$\text{expression pattern divergence} \sim \text{network conservation category} + \text{phylogenetic distance} \tag{26}$$

Based on the model, there is no significant difference in expression pattern divergence between regulators with conserved and diverged modules ($\beta = -0.16$, $p = 0.33$).

As a complementary analysis, we also fitted the following linear model across all regulators (for non-redundant species comparisons only):

$$\text{expression pattern divergence} \sim \text{network divergence (residual)} + \text{phylogenetic distance} \tag{27}$$

The continuous measure of network divergence (residual) was also not a significant predictor ($\beta = -0.49$, $p = 0.12$).

Association between the *POU5F1* module and LTR7 elements

LTR7 elements in the human genome were defined based on intersection of the annotation reported by Ito *et al.* and the annotation created with RepeatMasker [135]. Information on ChIP-seq-based POU5F1 binding and lineage specificity was also taken from Ito *et al.*. LTR7 elements were associated with genes based on distance, using a ±100 kb search space around the TSSs. Since LTR7 elements are known to be active during pluripotency, we restricted the analysis to genes expressed in human iPSCs (detected in at least 10% of iPSCs with a mean log-normalized expression level > 0.1). *POU5F1* module member genes were significantly more often linked to one or more LTR7 elements than other expressed genes (Fisher's exact test, odds ratio = 3.20, $p = 0.005$). This enrichment was even stronger for LTR7 elements containing POU5F1 binding sites (odds ratio = 10.6, $p = 0.0002$) and for LTR7 elements lacking an ortholog in the cynomolgus macaque genome (odds ratio = 8.99, $p = 0.001$).

For Additional File 1: Fig. S12–S14, LTR7 and HERVH-int elements at the *SPP1* and *SCGB3A2* loci were identified in the gorilla and cynomolgus macaque genomes using RepeatMasker [135].

3D genome architecture upstream of *SPP1*

To investigate chromatin interactions upstream of *SPP1*, we used published high-throughput chromosome conformation capture (Hi-C) data from human and cynomolgus macaque. The experimental procedures and the processing of these data are described in detail in the original publication [61]. Briefly, *in situ* Hi-C libraries were generated using a modified version of the protocol introduced by Rao *et al.* [136]. Reads were mapped to the hg38 and macFas6 reference genomes and post-processed using Juicer [137], then the contact frequencies were normalized using Shaman [138]. kNN-based Hi-C scores [139] were calculated for all replicates of a species combined, with the kNN parameter set to 100. The aligned synteny was plotted with SVbyEye [140].

Supplementary References

120. Dobin, A., Davis, C. A., Schlesinger, F., *et al.* STAR: ultrafast universal RNA-seq aligner. *Bioinformatics* **29,** 15–21 (2013).

121. HUGO Gene Nomenclature Committee (HGNC), European Molecular Biology Laboratory, European Bioinformatics Institute (EMBL-EBI), Wellcome Genome Campus, Hinxton, Cambridge CB10 1SD, United Kingdom. HGNC Database. `www.genenames.org`. Accessed: 2023-5-12.

122. Durinck, S., Moreau, Y., Kasprzyk, A., *et al.* BioMart and Bioconductor: a powerful link between biological databases and microarray data analysis. *Bioinformatics* **21,** 3439–3440 (2005).

123. McCarthy, D. J., Campbell, K. R., Lun, A. T. L., *et al.* Scater: pre-processing, quality control, normalization and visualization of single-cell RNA-seq data in R. *Bioinformatics* **33,** 1179–1186 (2017).

124. Csárdi, G. & Nepusz, T. The igraph software package for complex network research. *InterJournal Complex Systems* **1695,** 1–9 (2006).

125. Li, H. Minimap2: pairwise alignment for nucleotide sequences. *Bioinformatics* **34,** 3094–3100 (2018).

126. Langmead, B., Trapnell, C., Pop, M., *et al.* Ultrafast and memory-efficient alignment of short DNA sequences to the human genome. *Genome Biol.* **10,** R25 (2009).

127. Zhang, Y., Liu, T., Meyer, C. A., *et al.* Model-based analysis of ChIP-Seq (MACS). *Genome Biol.* **9,** R137 (2008).

128. Amemiya, H. M., Kundaje, A. & Boyle, A. P. The ENCODE Blacklist: Identification of Problematic Regions of the Genome. *Sci. Rep.* **9,** 9354 (2019).

129. Huang, X. & Huang, Y. Cellsnp-lite: an efficient tool for genotyping single cells. *Bioinformatics* **37,** 4569–4571 (2021).

130. Huang, Y., McCarthy, D. J. & Stegle, O. Vireo: Bayesian demultiplexing of pooled single-cell RNA-seq data without genotype reference. *Genome Biol.* **20,** 273 (2019).

131. Ritchie, M. E., Phipson, B., Wu, D., *et al.* limma powers differential expression analyses for RNA-sequencing and microarray studies. *Nucleic Acids Res.* **43,** e47 (2015).

132. Malta, T. M., Sokolov, A., Gentles, A. J., *et al.* Machine Learning Identifies Stemness Features Associated with Oncogenic Dedifferentiation. *Cell* **173,** 338–354 (2018).

133. Hao, Y., Stuart, T., Kowalski, M. H., *et al.* Dictionary learning for integrative, multimodal and scalable single-cell analysis. *Nat. Biotechnol.* **42,** 293–304 (2024).

134. Korsunsky, I., Millard, N., Fan, J., *et al.* Fast, sensitive and accurate integration of single-cell data with Harmony. *Nat. Methods* **16,** 1289–1296 (2019).

135. Smit, A. & Hubley, R. RepeatMasker. *Github.* `https://github.com/Dfam-consortium/RepeatMasker` (2017).

136. Rao, S. S. P., Huntley, M. H., Durand, N. C., *et al.* A 3D map of the human genome at kilobase resolution reveals principles of chromatin looping. *Cell* **159,** 1665–1680 (2014).

137. Durand, N. C., Shamim, M. S., Machol, I., *et al.* Juicer provides a one-click system for analyzing loop-resolution Hi-C experiments. *Cell Syst.* **3,** 95–98 (2016).

138. Cohen, N. M. The shaman package - Sampling HiC contAct Matrices for Aparametric Normalization. *Github.* `https://github.com/tanaylab/shaman` (2017).

139. Bonev, B., Mendelson Cohen, N., Szabo, Q., *et al.* Multiscale 3D genome rewiring during mouse neural development. *Cell* **171,** 557–572.e24 (2017).

140. Porubsky, D., Guitart, X., Yoo, D., *et al.* SVbyEye: a visual tool to characterize structural variation among whole-genome assemblies. *Bioinformatics* **41,** btaf332 (2025).
